# Microgels for Cell Delivery in Tissue Engineering and Regenerative Medicine

**DOI:** 10.1007/s40820-024-01421-5

**Published:** 2024-06-17

**Authors:** Leyan Xuan, Yingying Hou, Lu Liang, Jialin Wu, Kai Fan, Liming Lian, Jianhua Qiu, Yingling Miao, Hossein Ravanbakhsh, Mingen Xu, Guosheng Tang

**Affiliations:** 1 Guangzhou Municipal and Guangdong Provincial Key Laboratory of Molecular Target and Clinical Pharmacology, the NMPA and State Key Laboratory of Respiratory Disease, School of Pharmaceutical Sciences and the Fifth Affiliated Hospital, Guangzhou Medical University, Guangzhou, 511436 People’s Republic of China; 2 School of Automation, Hangzhou Dianzi University, Hangzhou, 310018 People’s Republic of China; 3 Department of Biomedical Engineering, The University of Akron, Akron, OH 44325 USA; 4 Wallace H. Coulter Department of Biomedical Engineering, Georgia Institute of Technology, Atlanta, GA 30332 USA

**Keywords:** Microgels, Cell delivery, Scaffolds, 3D bioprinting, Single-cell microgels

## Abstract

Microgels prepared from natural or synthetic hydrogel materials have aroused extensive attention as multifunctional cells or drug carriers, that are promising for tissue engineering and regenerative medicine. Microgels can also be aggregated into microporous scaffolds, promoting cell infiltration and proliferation for tissue repair. This review gives an overview of recent developments in the fabrication techniques and applications of microgels. A series of conventional and novel strategies including emulsification, microfluidic, lithography, electrospray, centrifugation, gas-shearing, three-dimensional bioprinting, etc. are discussed in depth. The characteristics and applications of microgels and microgel-based scaffolds for cell culture and delivery are elaborated with an emphasis on the advantages of these carriers in cell therapy. Additionally, we expound on the ongoing and foreseeable applications and current limitations of microgels and their aggregate in the field of biomedical engineering. Through stimulating innovative ideas, the present review paves new avenues for expanding the application of microgels in cell delivery techniques.

**Supplementary Information:**

The online version contains supplementary material available at 10.1007/s40820-024-01421-5.

## Highlights

- This review provides a comprehensive summary associated with recent progress in the preparation and application of microgels.
- The characteristics and applications of microgels and microgel-based scaffolds for cell culture and delivery are elaborated with an emphasis on the advantages of these carriers in cell therapy.
- This review expounds on the ongoing and foreseeable applications and current limitations of microgels and their aggregate in the field of biomedical engineering.
- Through stimulating innovative ideas, the present review paves new avenues for expanding the application of microgels in cell delivery techniques.

**Supplementary Information:**

The online version contains supplementary material available at 10.1007/s40820-024-01421-5.

## Graphical abstract

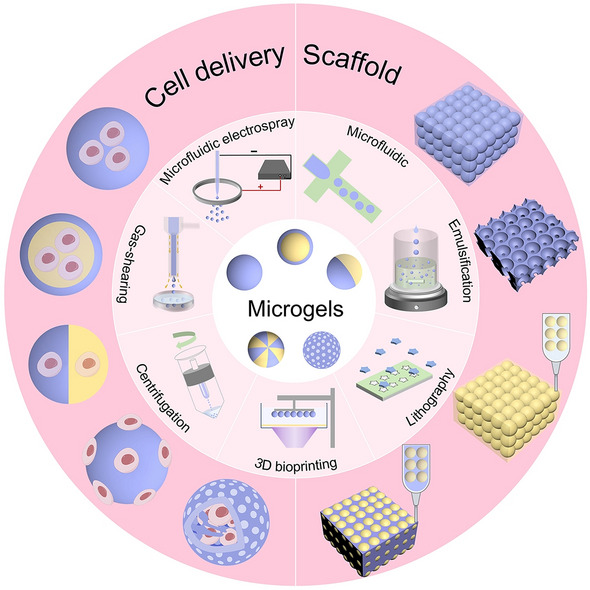

## Introduction

**Fig. 1:**
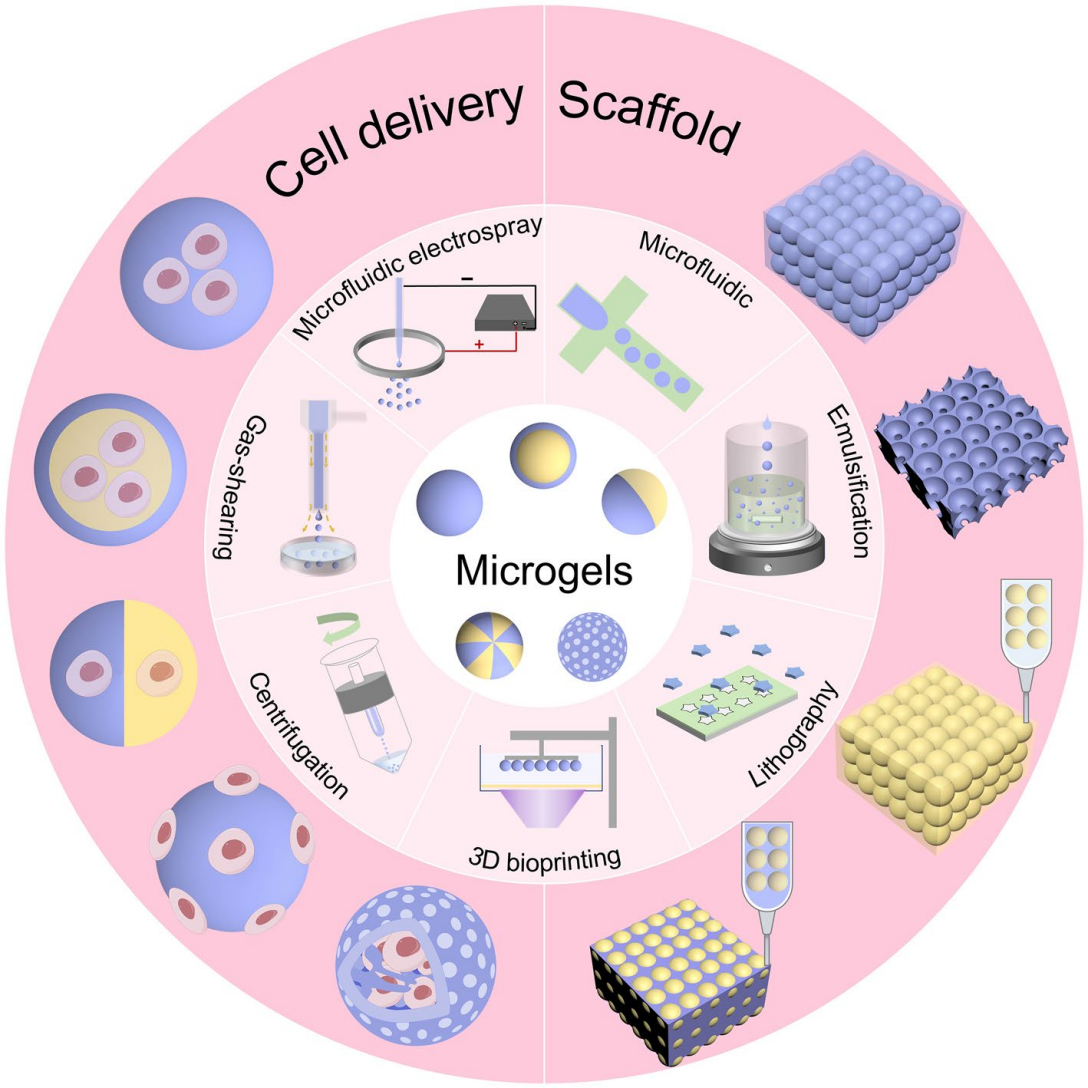
Preparation and application of microgels

**Fig. 2:**
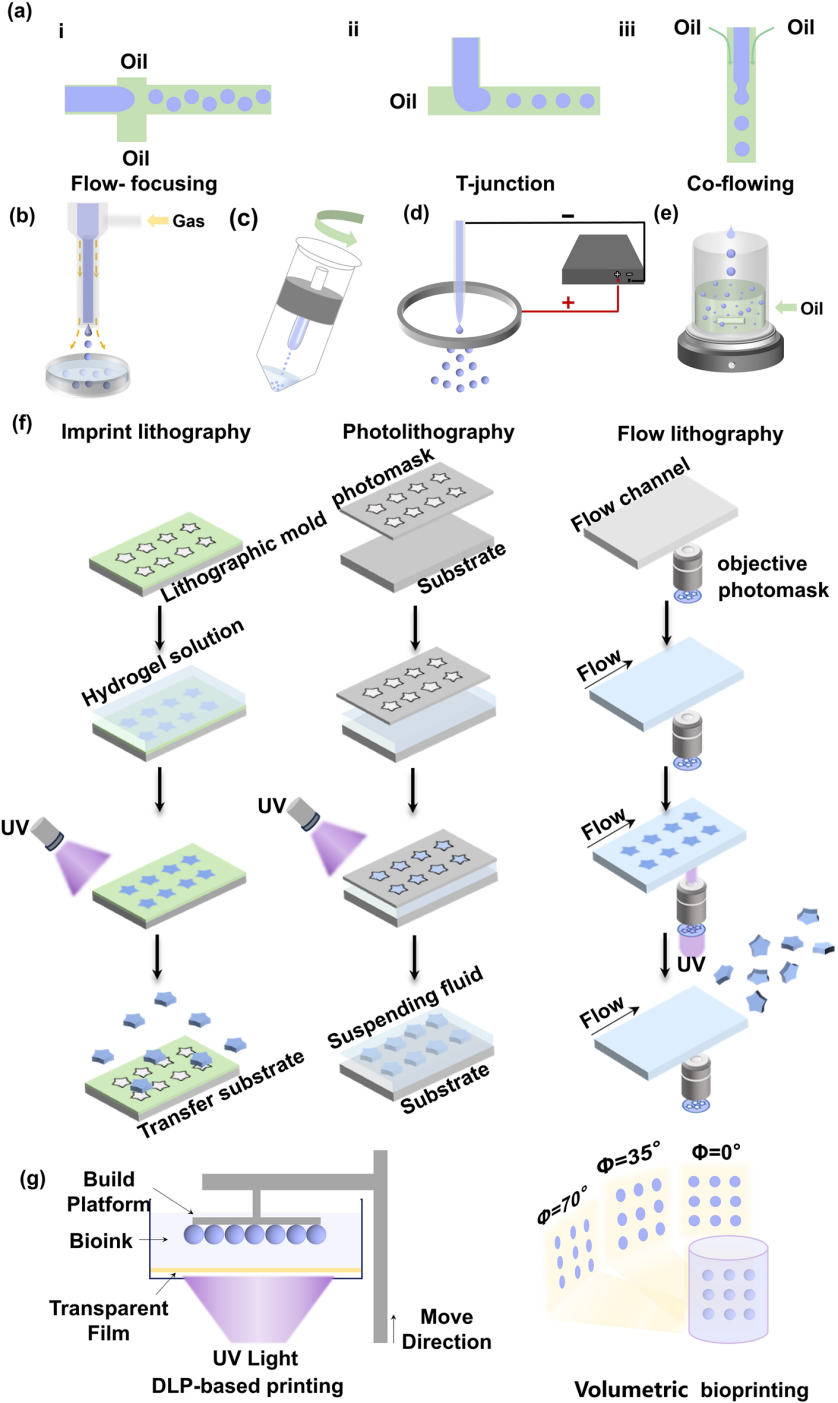
Fabrication methods of microgels. **a** Microfluidic technology. The flow-focusing (i), T-junction (ii), and co-flowing (iii) techniques are used to generate droplets that can be subsequently crosslinked to form microgels. **b** Gas-shearing technique. The shear force caused by the gas flow is employed to overcome the surface tension of the liquid making monodisperse droplets. The droplets are then crosslinked to form microgels. **c** Centrifugation. The hydrogel solution is extruded from the capillary via centrifugal forces. **d** Microfluidic electrospray. The electric field is used to form droplets. **e** Emulsification. Immiscible liquids are mixed and stirred to generate droplets. **f** Lithography. Photomasks are used to form microgels of uniform size and morphology. **g** 3D bioprinting. 3D bioprinting including DLP-based printing and Volumetric bioprinting are used for the fabrication of microgels

**Fig. 3:**
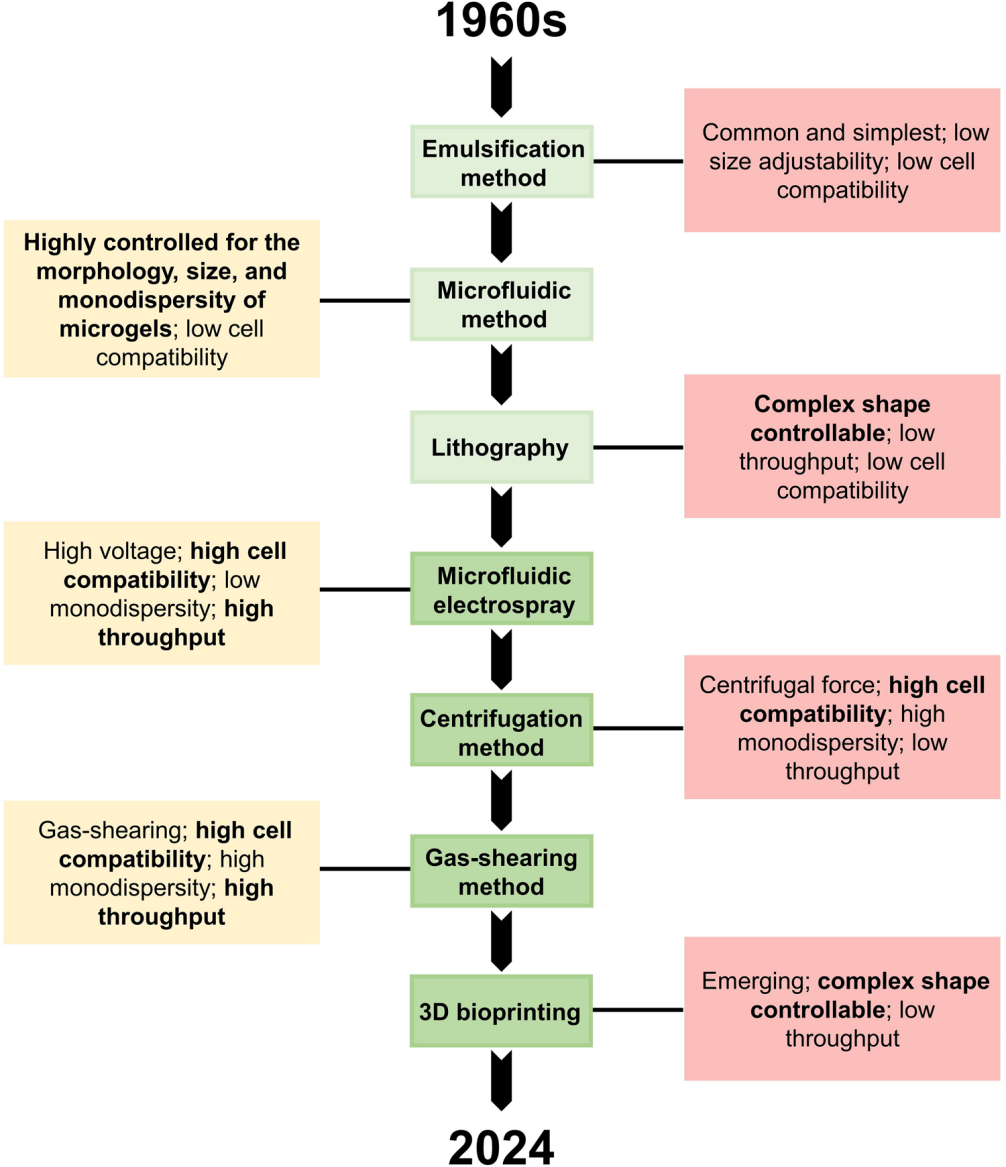
Characteristics of microgel fabrication techniques strategies

Hydrogels are a category of biopolymers derived from natural or synthetic products [1–3]. Due to their high water content, similar properties to the extracellular matrix, biocompatibility, and degradability, hydrogels have been utilized in a wide range of cell delivery applications [4–6] (Fig. 1). Initially, hydrogels were mostly used as whole blocks, but their application in cell delivery was significantly limited by nanoscale pores [7], which may hinder cell growth. To overcome this challenge, various new strategies have been explored to transform bulk hydrogels into micron-scale granular hydrogels (microgels) [8–12], such as emulsification [13], microfluidic technologies [6], lithography [11], electrospray technique [15], centrifugation-based methods [14], gas-shearing methods [16] and three-dimensional (3D) bioprinting [17] (Fig. 2). Microgel normally refers to micron-level (1–1000 μm) granular hydrogel (Fig. S1). It can be generated in a variety of morphologies according to application requirements under different strategies (Fig. 3). Traditional bulk hydrogel-based biomaterials mainly use simple diffusion for substance transfer, but their penetration distance is very limited, thus hindering cell growth and migration. Microgels facilitate the efficient encapsulation and transfer of nutrients and metabolic products, enhancing intercellular and cell–matrix interactions through their good injectability, porosity, and large specific surface area. Such unique characteristics make them a promising option for use as carriers in cell delivery. Microgels also have the advantages of small size and high flexibility, enabling the formation of microgel aggregates for tissue repair. Microgel-based scaffolds can achieve multiple-cell combination therapy while maintaining an independent microenvironment of cells.

Cell delivery is one of the important therapeutic methods in regenerative medicine and tissue engineering. The efficiency of cell loading in microgels depends on many factors, including the type of biomaterial, preparation technique, and crosslinking method. Microgels protect cells throughout the encapsulation and delivery process, necessitating a gentle fabrication method to ensure cellular integrity [18]. By contrast, adsorbing cells on the surface of microgels are typically carried out after preparation of the microgels to avoid cells damage during the fabrication process [19, 20]. Multicellular microgels delivery have proven its therapeutic effectiveness in various fields. However, single-cell microgels have certain advantages as a complementary strategy. For example, single-cell encapsulation increases cell entrapment surface area and volume ratio, which further improves material and molecular transport. Another advantage of single-cell microgel is endowing cells with higher flexibility to address some of the challenges that multicellular microgels cannot solve [21–23], such as single-cell sequencing, stem cell delivery by intravenous injection or the research on the metabolic mechanisms in individual cells.

The fabrication of biological scaffolds from cell-laden microgels is a pivotal endeavor in biomedical engineering. Scaffolds composed of microgels carrying cells or bioactive substances are considered to have great potential in tissue regeneration. They not only provide space for cell proliferation, migration, and differentiation but also release active factors to repair or regenerate target tissues. Since hydrogel networks mimic the 3D extracellular matrix environment with remarkable biocompatibility, cell-laden microgels are conducive to in vitro modeling via providing an excellent platform for disease mechanism research and treatment [11, 21, 24]. However, the efficient and biocompatible preparation of cell-laden microgels remains a long-term challenge. Various efforts have been made to develop novel microgel-based scaffold fabrication methods, with strategies encompassing the use of sacrificial microgels, 3D printing technologies, and refined centrifugation processes [25–28].

The present work focuses on the advancements in the fabrication of microgels for cell delivery in tissue engineering and regenerative medicine, which has been overlooked in previous reviews. We comprehensively elaborate the preparation techniques, main principles, characteristics, and biomedical applications of microgels and microgel-based scaffolds for cell delivery. By bringing the existing challenges under the spotlight, we discuss future perspectives of employing microgels and their aggregate in cell delivery for tissue engineering and regenerative medicine. We believe this review will accelerate the development of microgels and give inspiration to future biomedical research for multidisciplinary applications.

## Fabrication of Microgels

A variety of fabrication techniques for microgels have been proposed so far. According to the differences in the power source and the material characteristics, the microgel fabrication technologies have been mainly divided into six categories (Table 1). These methods are different in terms of biocompatibility, monodispersity, throughput, etc. Apart from the fabrication technique, the choice of crosslinking method (click crosslinking, photocrosslinking, ion crosslinking, or physical crosslinking) may also affect cell viability and functionality. In this section, we focus on the advantages, limitations, and suitability of each fabrication technology for cell or drug encapsulation.

**Table 1:** Advantages and disadvantages of the available methods for generating microgels

Method	Power source	Cell compatibility	Abundance of compatible polymers	Monodispersity	Throughput	Size adjustability
Emulsification method	Mixing power	Low	Low	Low	High	High
Microfluidic method	Oil cut	Low	High	High	High	High
Lithography	The negative pattern of the mask	Low	Low	High	Low	Low
Microfluidic electrospray	High voltage	Medium	High	Medium	High	High
Centrifugation method	Centrifugal force	Medium	Low	Medium	Low	Medium
Gas-shearing method	Gas-shearing	High	High	High	High	High
3D bioprinting	Layer-by-layer/volumetric printing	High	High	High	Low	Medium

### Emulsification

**Fig. 4:**
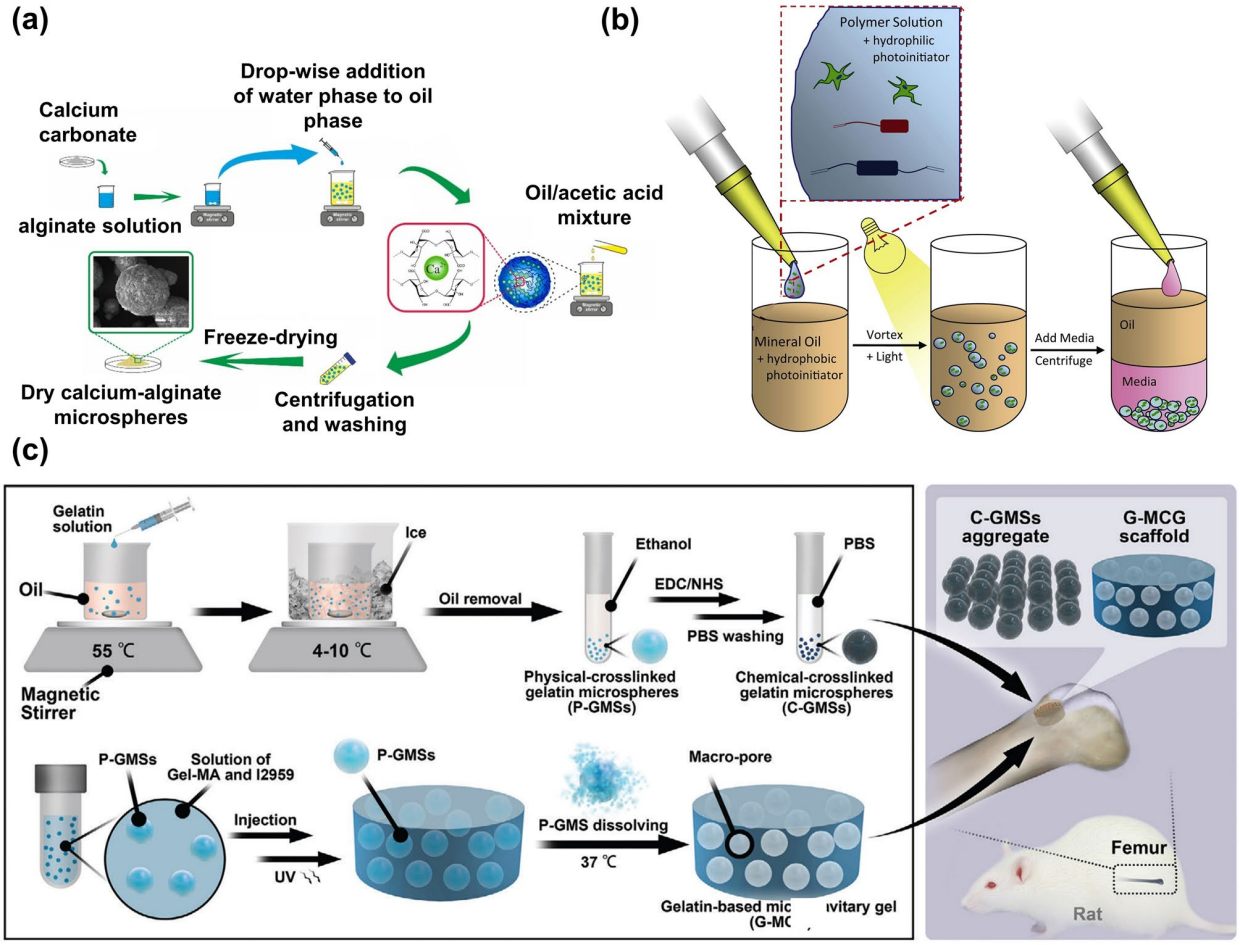
Preparation of microgels using the emulsification method. **a** Optimization of parameters for preparation of microgels by emulsification. Reproduced with permission [13]. Copyright 2020, Elsevier Science Ltd. **b** Uniform microgels prepared using a dual-initiator system, which contains a hydrophilic photoinitiator in the aqueous phase and a hydrophobic initiator in the oil phase. Reproduced with permission [29]. Copyright 2011, Elsevier Science Ltd. **c** Method of microgels fabricated by emulsification method to construct 3D scaffolds and promote angiogenesis. Reproduced with permission [27]. Copyright 2022, Wiley

Emulsification is one of the most common and simplest methods for generating microgels. Generally, the hydrogel precursor solution is mixed with immiscible oil to form droplets, and surfactants are added to the emulsion system to stabilize the emulsion. The morphology of the microgels generally depends on the polymer concentration (low concentration yields irregular microgels and high concentration yields spherical microgels), emulsification rate, and water/oil volume ratio. The surfactant concentration is a key parameter controlling the emulsion stability and microgel size (Fig. 4a) [13]. An increase in surfactant concentration typically results in a reduction in the average size of the microgels. The emulsification method is easy to operate without using complicated instruments while possessing a high throughput efficiency. However, it is still a challenge to control the morphology and monodispersity of microgels using the emulsion method. Normally, hydrogel prepolymers containing a photoinitiator or crosslinker are photo-crosslinked into microgels. Instead of using a single photoinitiator, one study had shown that using a dual-photoinitiator could obtain microgels with a better morphology (Fig. 4b) [29]. Controlling or limiting the size range of microgels could be achieved by mechanically filtering the microgels or adding a stabilizing surfactant during synthesis.

In addition to preparing microgels with single emulsions strategy, there are also other approaches with double emulsions and multiple emulsions strategies to fabricate multicompartmental microgels. The structure of droplets could be readily transformed from encapsulation to Janus configuration via using hydrocarbons and fluorinated surfactants to change the interfacial tension [30]. Since microgels are easily obtained with high throughput efficiency from the emulsion method, it is often used in the preparation of scaffolds. Moreover, porous scaffold materials could be obtained through sacrificing microgels generally from, but not limited to, the emulsion method. For example, by removing chitosan microgels, calcium phosphate scaffolds were fabricated for bone tissue engineering [31]. Gelatin-based scaffolds with controllable pore structures could be also prepared by the emulsion template method (Fig. 4c) [27]. The fabricated scaffolds demonstrated an excellent ability to promote vascularized bone regeneration and integration into the host bone.

Although the inevitable use of oils and surfactants increases cytotoxicity, the emulsification method is also carried out to generate cell-laden microgels. For example, Lipke and coworkers proposed the encapsulation of cancer cells into microgels to establish tumor spheroid models for the study of cancer treatment and drug screening [32]. Tian et al. prepared sodium alginate (SA) microgels containing mesenchymal stem cells (MSCs) and magnetic nanoparticles with different concentrations by emulsification and assembled them to create a magnetically actuated micro-robot [33]. Under the electromagnetic effect, the micro-robot was flexible to target delivery MSCs. In general, controlling the microgel size is still a difficult challenge in the emulsion method. The heterogeneity of microgel size not only leads to poor repeatability between batches but also leads to differences in package contents, including the number of cells, drug content, etc. Therefore, the fabrication techniques of microgel with controllable size are still needed.

### Microfluidic Techniques

Droplet generation using microfluidic devices has been studied since the beginning of the twenty-first century [34–36]. Briefly, a microfluidic device usually introduces one fluid into another immiscible or partially immiscible fluid, which results in the formation of droplets at the meeting junction through the shearing force. Device structures including T-junction [37, 38], flow focusing [39], and co-flow [40] are commonly used to fabricate hydrogel droplets or microgels.

Droplet microfluidics technology has been extensively used for fabricating microgels, as it allows the highest control over the morphology, size, and monodispersity of microgels without changing the configuration of the microfluidics device. The size and morphology of the microgel depend on the flow rate ratio of the two fluids and the geometry of the channel, such as its width. Recently, cell-laden hydrogels have attracted considerable attention due to their unique 3D network, which is suitable for cell culture. For example, Utech et al. reported a method to prepare monodisperse sodium SA microgels with a uniform structure through droplet microfluidics [41]. By controlling the crosslinking process, highly homogenous SA microgels with reliable and precisely tunable particle properties were fabricated. Using this approach, microgels with diameters ranging from 10 to 50 µm were readily obtained. MSCs were grown and stably proliferated in the generated microenvironment while maintaining a high survival rate after 15 days.

**Fig. 5:**
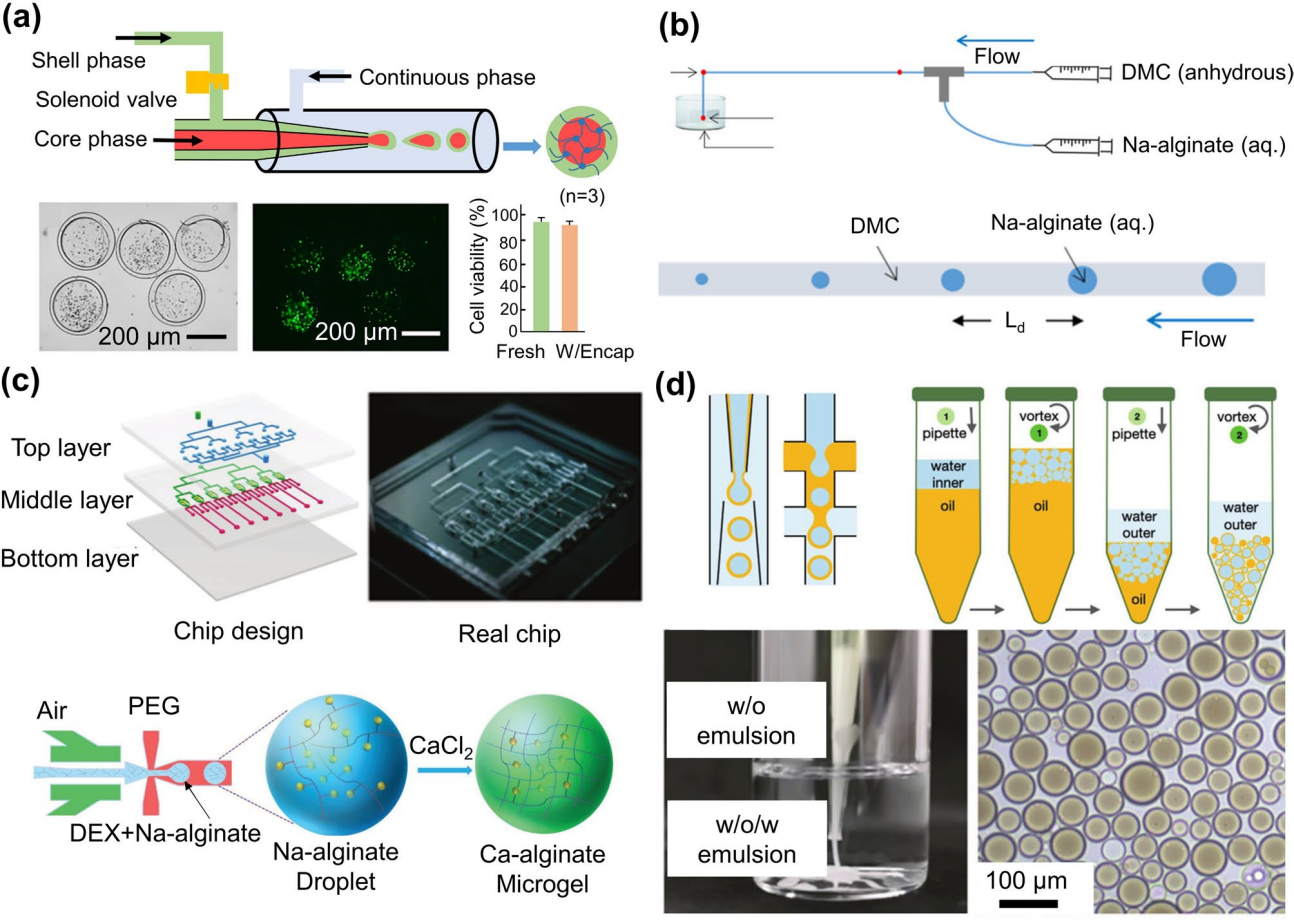
Microfluidic technique for the preparation of microgels. **a** Generation of core–shell microgels via all aqueous-phase microfluidic systems for 3D cell culture. Reproduced with permission [42]. Copyright 2019, American Chemical Society. **b** Fabrication of small-sized microgels that mimic red blood cells using microfluidic techniques. Reproduced with permission [43]. Copyright 2020, Pergamon-Elsevier Science Ltd. **c** Controllable and high-throughput generation of monodisperse water in water (W/W) droplets with a microfluidic chip. Reproduced with permission [44]. Copyright 2018, Wiley–VCH Verlag GmbH. **d** Preparation of single-core, multiple core–shell microgels with oil in water (W/O) or water in oil in water (W/O/W) emulsions. Reproduced with permission [45]. Copyright 2022, Wiley–VCH Verlag GmbH

Oil and surfactants are inevitably used in most microfluidic systems to form droplets. However, employing oil makes its subsequent removal troublesome, not to mention that the washing step will cause cell damage and may adversely affect cell viability. To address this challenge, Zhao and coworkers used poly (ethylene glycol) as the continuous phase, the mixture of SA and glucan as the shell phase, and cell-suspended sodium carboxymethylcellulose as the core phase to form cell-laden microgels (Fig. 5a) [42]. The microgels prepared by this all-aqueous-phase microfluidic system could be used in 3D cell culture, and the encapsulated cells maintained high viability.

The smaller microgels could be generated by exploiting the shrinkage properties of the material. Zhang et al. generated smaller microgels to simulate red blood cells by microfluidic techniques [43]. The water contained in the prepared microgels slowly diffused into the continuous phase along the microfluidic channel, resulting in reduced microgel size (Fig. 5b). The smaller microgels could be generated with a longer channel due to the higher shrinkage rate. However, this narrow microchannel often increases the risk of blockage, resulting in a relatively low reliability. In a later work, a microfluidic chip was designed for controllable and high-throughput generation of monodisperse water in water droplets to address this issue (Fig. 5c) [44]. The droplet microfluidic chip was composed of a control unit, a droplet generation unit, and a collection unit. In the absence of any oil and surfactant, the droplet production rate was ≈ 100 Hz in the 8-channel microfluidic device, which was higher than that of most aqueous systems.

In recent years, researchers have combined microfluidics with other microgel preparation methods, such as emulsification (Fig. 5d) [45]. Such techniques can be utilized to fabricate microgels with complex structures via changing microfluidic devices. Over the past few decades, the simple core–shell structure of microgels has expanded to materials with versatile compartments, compositions, morphology, and properties [46]. Highly uniform multiple-holed hollow microgels can be fabricated fast and straightforwardly via an off-the-shelf microfluidic device [47]. However, the inevitable use of oils and surfactants increases cytotoxicity, which remains a major restriction in microfluidic techniques, specifically when sensitive biological molecules or cells have to be encapsulated in microgels. Thus, replacing the oil phase with a biocompatible and thermodynamically incompatible aqueous phase has a high potential in biomedical applications. Besides, to obtain microgels with small sizes, most microfluidic channels are narrow, which applies shear stress on the cells and greatly reduces cell survival and bioactivity. Removing the restriction of channels using strategies, such as flow lithography, is also a prospective direction for the development of microgel fabrication.

### Lithography

In general, the morphology of the microgels can be controlled by parameters such as oil/gas and polymer flow rates, and the width of the microfluidic channels [48]. However, the morphology of microgels is always limited to spherical or sphere-based geometries. Lithography, a method that can accurately control the morphology and size of microgels, provides the morphology of microgels a huge plasticity through the negative mask with different shapes. The hydrogel precursor solution with the photoinitiator is introduced into the PDMS mold with spatially controlled ultraviolet exposure. The microgels are then solidified to obtain the morphology corresponding to the mask [49, 50].

Lithography is further subdivided into imprint lithography, photolithography, and flow lithography. In imprint lithography, materials with poor fluidity and wettability cannot fill the mold, which will reduce the morphological integrity of microgels. A simple degassed micromolding lithography was proposed to successfully solve this problem [51]. This technique involves degassing the mold within a vacuum chamber before synthesizing the microgels. The degassed mold then acts as a suction pump, effectively eliminating air bubbles between the fluid and the mold, thus ensuring the fluid conforms precisely to the mold's patterning. This strategy has significantly improved the resolution and integrity of microgel structures created by imprint lithography.

Flow lithography has several advantages, including high production efficiency, versatility of structures, and simple operation. Flow lithography allows two or more fluids to enter the same channel at the same time. Each fluid can maintain its flow pattern, while molecules only diffuse at the adjacent interface. Therefore, the characteristics of laminar flow, such as stable flow velocity and no macroscopic mixing between fluids provide a unique platform for the manufacturing of microgels composed of different materials with complex morphologies and functions [52]. Compared to microfluidic strategies, flow lithography also addresses the problem of low cell activity due to narrow microfluidic channels.

**Fig. 6:**
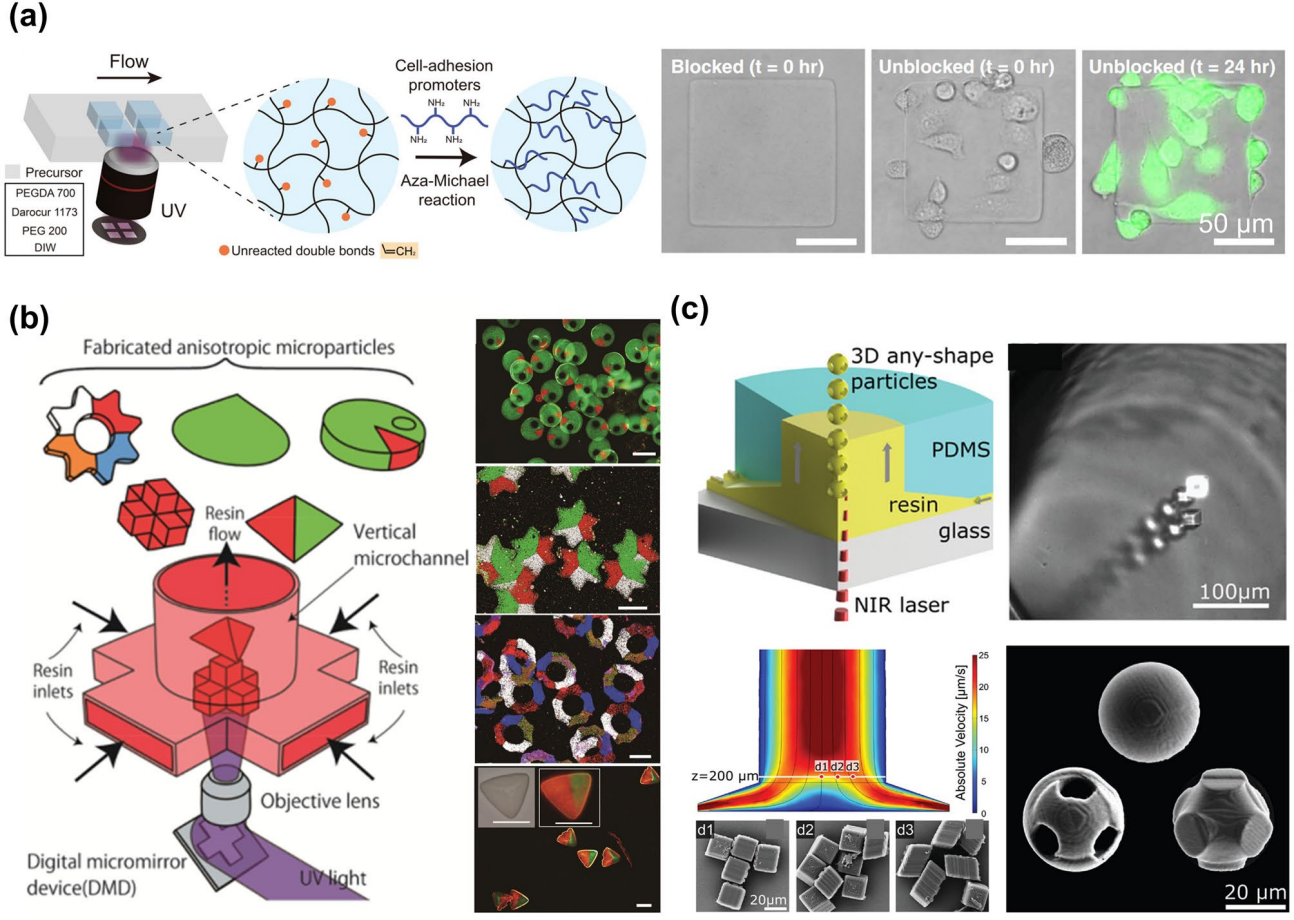
Illustration of microgels by lithography. **a** Strategy of microgel via stop-flow lithography for increasing the number of adherent cells. Reproduced with permission [53]. Copyright 2022, Wiley. **b** Schematic and photograph of complex anisotropic microparticles by vertical flow lithography with angular segmented flow formed by various resins. Reproduced with permission [57]. Copyright 2015, Wiley–VCH Verlag GmbH. **c** Fabrication of anisotropic microparticles via two-photon vertical flow lithography. Reproduced with permission [59]. Copyright 2022, Wiley–VCH Verlag GmbH

To improve the biocompatibility of cell-laden microgels, cell attachment can be performed after the synthesis of microgels. However, cell-attachment strategies often require additional linker molecules and complicated pre-conjugation steps. To address these problems, polyethylene glycol microgel produced by stop-flow lithography was directly functionalized with poly-L-lysine and Gly-Arg-Gly-Asp-Ser peptides (Fig. 6a) [53]. This method was a simple but effective strategy to achieve cell adhesion on the microgels and significantly increase the number of adherent cells.

Apart from hydrogels, other materials are also widely used to fabricate microparticles with complex structures. It is worth mentioning that multicompartmental microparticles have attracted considerable attention in the biomedical field because a single microparticle can carry multiple materials with different components well separated from each other to achieve versatilities [54–56]. When a variety of resins were used, it was possible to produce angularly segmented flows distinct from laminar flow, forming microparticles with separate compartments (Fig. 6b) [57].

Recently, to further improve the controllability of microparticle morphology and size, two-photon photolithography combined with light-induced deoxyribonucleic acid (DNA) has been widely used to prepare complex microparticles, such as tetrahedron, pyramid, and other polyhedral structures [50, 58]. The ratio of surface to volume is the main factor in the action of certain cells, enzymes, or microorganisms. Microparticles smaller than 100 μm, with complex morphologies and high resolution can be manufactured continuously via two-photon vertical flow lithography (Fig. 6c) [59].

Although the lithography method can be employed for fabricating various morphologies of microgels through the mold, there are limitations associated with this technique. Using the mold limits the complexity of the external or internal structure of the microgels and also leads to low throughput. More importantly, due to the harsh process conditions and limited candidate materials, the controllable delivery of the cells via microgels in various biomedical fields is still restricted. Therefore, it has always been a challenge to fabricate microgels with high cell viability using the lithography method. In summary, highly biocompatible materials are still required to be developed for lithography.

### Microfluidic Electrospray

Microfluidic electrospray has been widely used in the fabrication of microgels for drug delivery and cell encapsulation due to its favorable biocompatibility. In previous studies, this technique has been mostly used to fabricate small-size microgels for drug delivery [60, 61], but the fabricated microgels have less monodispersity. With the advancement of equipment and technology, the control of microgels using the microfluidic electrospray method is becoming increasingly precise [62]. In the microfluidic electrospray technique, a hydrogel precursor solution is extruded through a syringe while generating droplets from the needle tip under an external electric field. The size of droplets is influenced by the applied voltage, properties of hydrogels, polymer flow rate, and concentration, nozzle size, etc. [63]. The morphology of the microgels could be optimized by adjusting the distance between the nozzle and the collector [64].

**Fig. 7:**
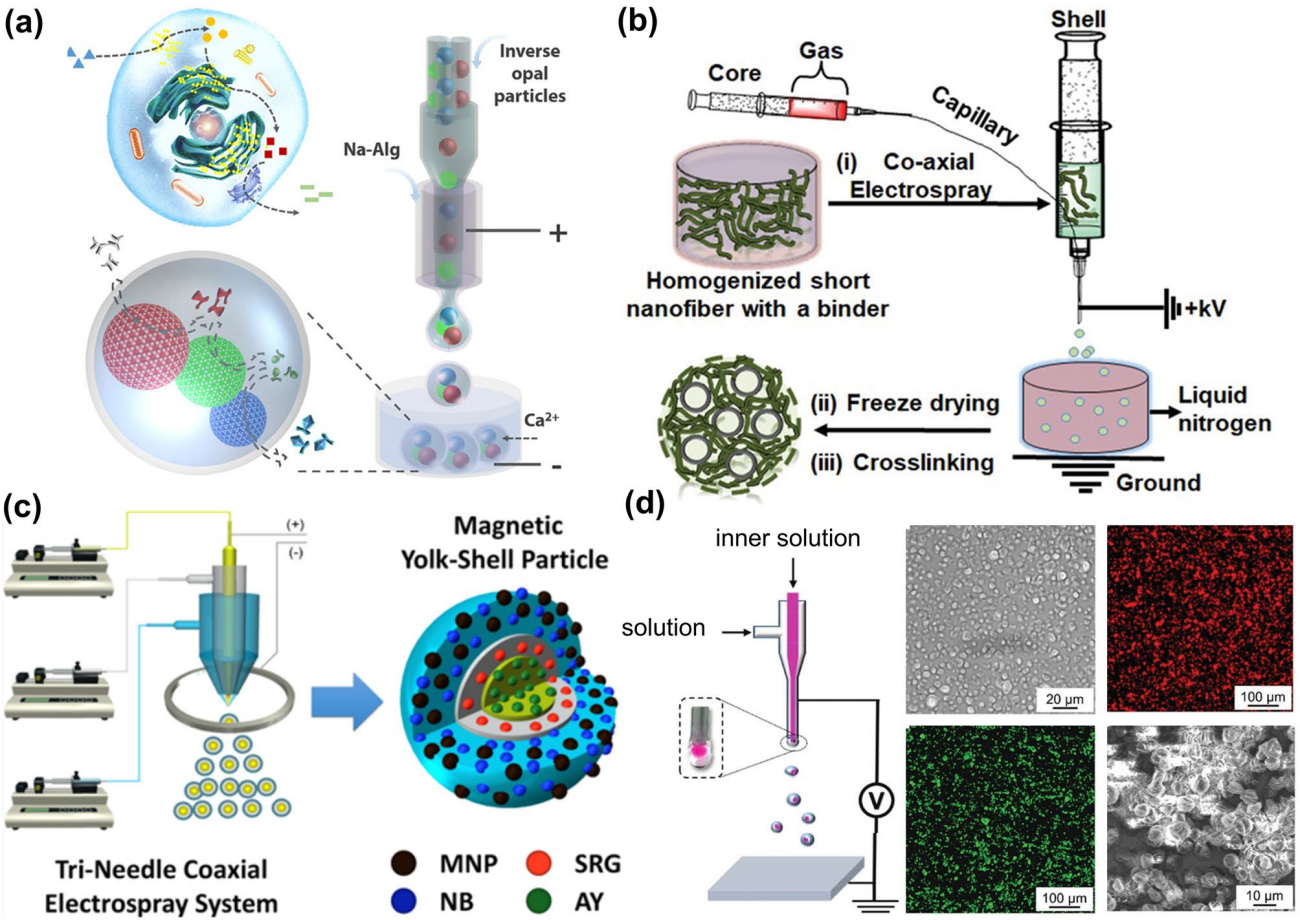
Examples of microgels by microfluidic electrospray. **a** Schematic of microgels with multiple cores for biomimetic enzyme cascade reaction. Reproduced with permission [68]. Copyright 2018, American Association for the Advancement of Science. **b, c** Fabrication of porous microgels **b**, and spherical core–shell microgels **c** via microfluidic electrospray. Reproduced with permission [65]. Copyright 2020, Wiley–VCH Verlag GmbH [71]. Copyright 2017, Wiley–VCH Verlag GmbH. **d** Preparation of core–shell microgels combined with a near-infrared laser photothermal heating for releasing nerve growth factor and promoting neurite outgrowth. Reproduced with permission [76]. Copyright 2018, Wiley–VCH Verlag GmbH

Although the microgels size can be as small as a micron scale, obtaining monodisperse microgels is challenging when the microfluidic electrospray technique is employed. Nonetheless, monodisperse microgels could be achieved using filters. Furthermore, Zhao and coworkers proposed a series of strategies to generate monodisperse microgels, which have been used in various fields such as bone regeneration, organoids, and biomimetic enzyme cascade reaction (Fig. 7a) [15, 65–68]. Combined with inverse opal particles containing an ordered macroporous structure, the microcapsules could effectively control the number, type, and arrangement of the encapsulated enzymes. The multi-enzyme system mimics hepatocytes and performs elementary alcohol detoxification.

The microgels prepared by microfluidic electrospray are excellent carriers for cell delivery as there is no oil or surfactant included in the fabrication process. Wang et al. mixed cells into the precursor hydrogel solution to prepare SA porous microgels encapsulated with natural killer cells [69]. While protecting cells from the surrounding environment, microgel could continuously secrete perforin and granzymes which exhibited robust killing effects on tumors. Microfluidic electrospray can encapsulate not only cells for disease treatment but also cancer cells for drug evaluation. Zhao’s group encapsulated primary human pancreatic cancer cells in hydrogel microgels composed of SA and carboxymethyl cellulose (CMC) [65]. The cells maintained high bioactivity after encapsulation and proliferated rapidly to form 3D biomimetic tumor spheroids with highly uniform sizes.

The microfluidic electrospray technique can be also used for fabricating a variety of microgels with different structures, including porous microgels [64], core–shell microgels [68, 70], and microfiber-laden microgels [71] (Fig. 7b, c). Microfluidic electrospray, as a multifunctional technology, holds great promise in the field of regenerative medicine. Particularly, microgels made through microfluidic electrospray are used for tumor treatments [72, 73], bone regeneration [74], endodontic regeneration [75], and neurite outgrowth (Fig. 7d) [76]. However, when microgels with smaller sizes need to be prepared, stronger electric fields have to be used, which would inevitably reduce cell bioactivity and monodispersity. Changing the type of current (alternating current and direct current) may help to improve the stability and monodispersity of microfluidic electrospray technology [77].

### Centrifugation-Based Method

**Fig. 8:**
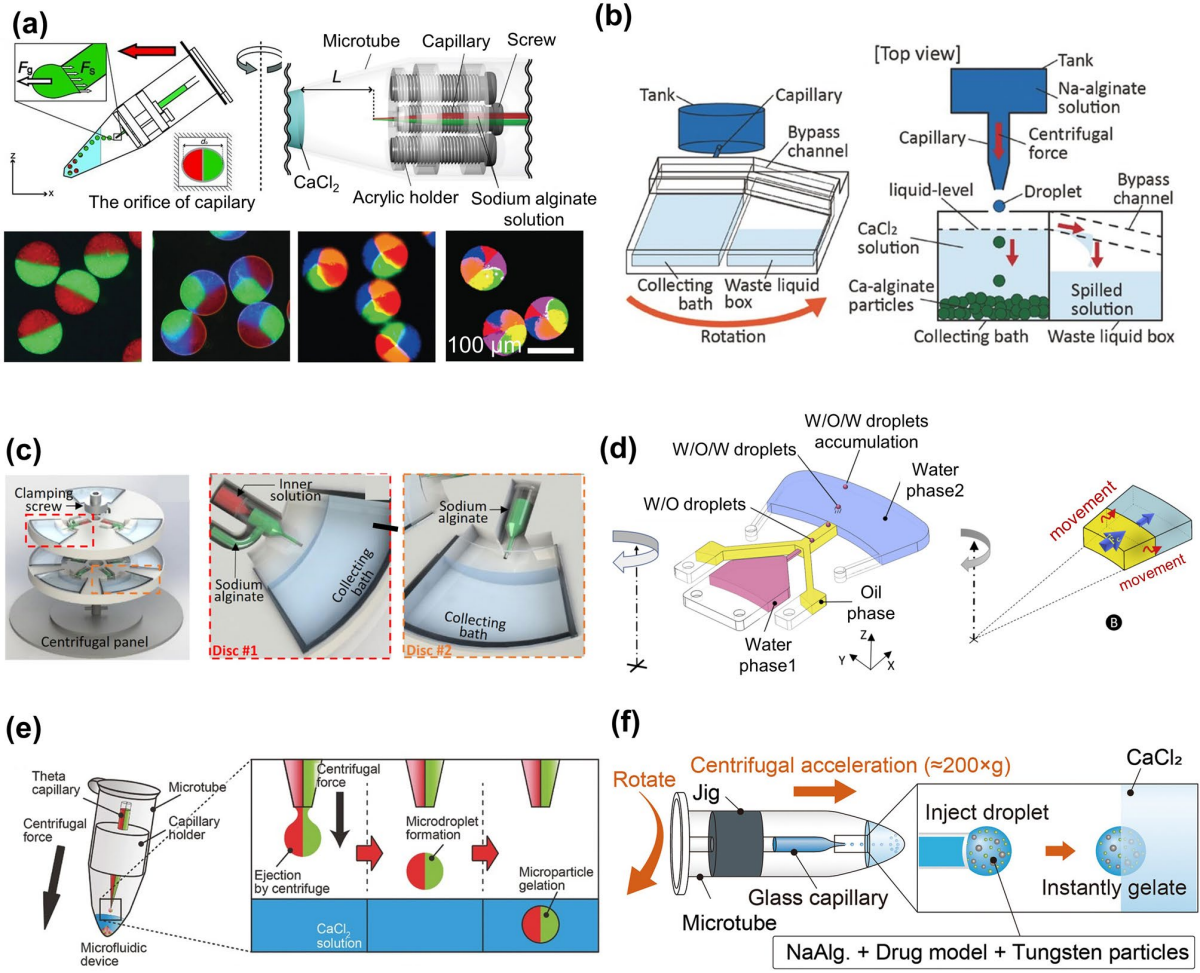
Centrifugation-based methods for the preparation of microgels. **a** Multi-barrelled capillary technique. Reproduced with permission [78]. Copyright 2012, Wiley–VCH Verlag GmbH. **b, c** Fabrication of microgels in a high-throughput fashion by adding a waste liquid box (**b**) and multiple nozzles (**c**). Reproduced with permission [79]. Copyright 2017, Elsevier Science SA [81]. Copyright 2018, Springer Heidelberg. **d** Schematic of size-controlled and uniform microgels by adding an oil barrier for controlling the crosslinking process between sodium alginate and Ca^2+^. Reproduced with permission [82]. Copyright 2020, SAGE Publications Inc. **e** Preparation of multi-compartment microgels and selectively encapsulate cells in different compartments. Reproduced with permission [83]. Copyright 2017, Wiley. **f** Schematic showing drug-loaded microgels via centrifugation method for ultrasound-triggered drug administration. Reproduced with permission [84]. Copyright 2021, Elsevier

Microgels can be fabricated by centrifugal forces in a controllable fashion without using oil and surfactant. In this technique, the SA solution is extruded from the capillary via centrifugal forces and forms microdroplets, then immediately solidified in CaCl_2_ solution (Fig. 8a) [78]. The morphology and size of microgels are controlled by the surface tension, viscosity of the hydrogel precursor solution, and capillary nozzles. High throughput fabrication of microgel could be achieved by increasing centrifugal force. However, with the increase of microgels, the receiving bath level continues to rise, which changes the distance between the nozzle and the liquid surface of the collecting bath, resulting in the deformation of the microgels. Hence, centrifuge-based systems cannot be used to generate microgels with high monodispersity or high throughput.

As shown in Fig. 8b, a waste liquid box, which can receive excess liquid next to the collecting bath, was employed to address this problem [79]. Using this approach, the distance between the nozzle and the liquid level remained constant during a certain time. Increasing the number of nozzles can also improve the throughput of microgels. The centrifuge system with multiple nozzles can stably and controllably form microgels with different structures at the same time [14, 80]. Besides simple structured microgels, hydrogel fibers, and core–shell structured capsules/fibers can be formed by changing the structure of the nozzle and the distance between the nozzle and the liquid level (Fig. 8c) [81]. As another improvement for preventing the rapid interaction of SA with Ca^2+^, an oil barrier was also introduced in the device to control the crosslinking process of microgels while producing water-in-oil-in-water emulsions (Fig. 8d) [82].

Since the centrifugal method is relatively biocompatible without using oils and surfactants, this technique has become a platform for a variety of applications, such as cell encapsulation, and drug delivery. Under centrifugation, cells can be selectively encapsulated in compartments of extracellular matrix (ECM)-based microgels with high viability (Fig. 8e). For example, Hasturk et al. proved the preservation of the bioactivity and differentiation capacities of both human mesenchymal stem cells (hMSCs) and human neural progenitor cells (hNPCs) after encapsulation [83]. Centrifugation combined with ultrasound-triggered drug administration can effectively improve the efficacy of drug therapy. For instance, adding tungsten microgels with high acoustic impedance into the hydrogel precursor solution increased the sensitivity of the microgels to ultrasound and promoted drug release [84]. The microgel coating with poly-L-lysine can also prevent drug leakage and control drug release, thus achieving better drug efficacy (Fig. 8f). Although the centrifugation-based method is simple to operate and does not involve substances with low biocompatibility, the limitation of replacing the capillary nozzles to achieve size controllability cannot be overlooked. In view of this, the centrifugation device could be improved through different ways, such as altering the design of nozzles with continuous and controllable structures.

### Gas-Shearing Method

**Fig. 9:**
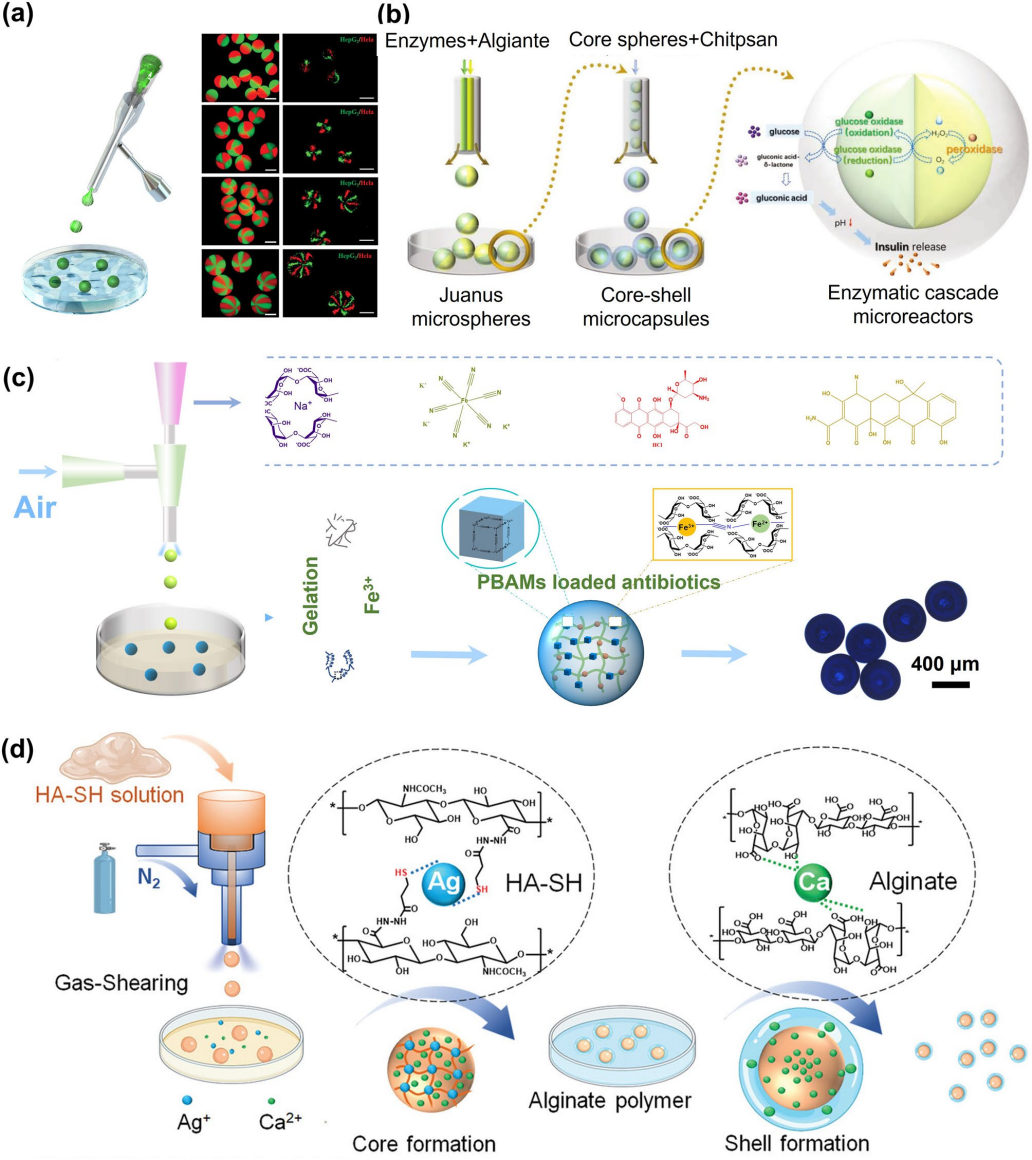
Preparation of microgels using the gas-shearing method. **a** Multicompartmental microspheres. Reproduced with permission [16]. Copyright 2019, Wiley. **b** Microgels loaded by glucose oxidase and catalase to simulate islet β cells in enzymatic cascade reactions. Reproduced with permission [87]. Copyright 2022, Wiley. **c** Microgels as carriers of drug for slowly releasing Prussian blue and achieving anti-tumor and antibacterial effects in photothermal therapy. Reproduced with permission [88]. Copyright 2022, Elsevier. **d** Core–shell microgels for the treatment of inflammatory bowel disease by targeting colon administration. Reproduced with permission [89]. Copyright 2021, Wiley

The gas-shearing process is a one-step, oil-free, photoinitiators-free, and surfactant-free approach with higher biocompatibility [85]. The device used for the gas-shearing technique introduces the liquid into the inner tube, while gas is transmitted through the space between the needle and the shell, generating a shear force that facilitates the formation of droplets. Apart from its high biocompatibility, the gas-shearing process can accurately control the size of droplets by adjusting the gas flow rate and nozzle size to generate microgels with sizes ranging from tens of microns to millimeters. In addition, the production efficiency of the microgels is readily controlled by adjusting the liquid flow rate. The production efficiency and morphology of the microgels are also affected by the liquid flow rate, collecting distance and angle, and solution concentration. Moreover, the gas-shearing approach is highly versatile and inclusive in the selection of materials including SA, chitosan, cellulose-acetate, cellulose-acetate-phthalate, etc. Also, microencapsulated multi-compartment microgels (MCMs) with up to eight compartments are easily prepared (Fig. 9a) [16]. More importantly, the gas-shearing strategy can be easily used to obtain multiple microenvironments within a single microgel to precisely assemble different cell types within a confined micrometer-sized volume. For example, Tang et al. demonstrated that Hela and human hepatocarcinoma (HepG_2_) can be encapsulated and co-cultured in the various compartments of one single microgel and maintain the bioactivity of cells [16]. This unique feature proffers an effective strategy to study the complex interactions between different cells or be used for combined therapy with multiple cell types.

More complex microgel structures could also be fabricated by two-step gas shearing. For example, inspired by the structure of eukaryotic cells, Qu et al. fabricated MCMs through a gas-shearing method for enzymatic cascade reaction [86, 87]. Glucose oxidase and catalase were wrapped in two compartments of the alginate core separately. After the microgels were collected, they could be coated with chitosan by re-gas shearing, while insulin was loaded in the shell (Fig. 9b). This combination of multi-compartment structure and core–shell structure was conducive to the occurrence of enzyme cascade reaction.

The high porosity of the microgels facilitates cell and drug delivery. Zhang et al. designed a drug carrier with single-compartment microgels using gas-shearing technology, in which Prussian blue particles were encapsulated and provided a porous structure (Fig. 9c) [88]. Similarly, core–shell microgels can be easily prepared using the gas-shearing strategy. Cui and coworkers combined gas shearing and ionic diffusion techniques to prepare microgels with viscous shells for colon-targeted drug delivery (Fig. 9d) [89]. Core–shell microgels could also provide a protective shell for drugs in gastric juices [90]. In general, gas-shearing method has been extensively explored to fabricate microgels with different morphologies and structures, however, the fabrication of smaller microgels (< 50 μm) with good monodispersity is still a challenge, which may be addressed by improving the core–shell nozzle or optimizing the stability of the airflow.

### 3D Bioprinting

**Fig. 10:**
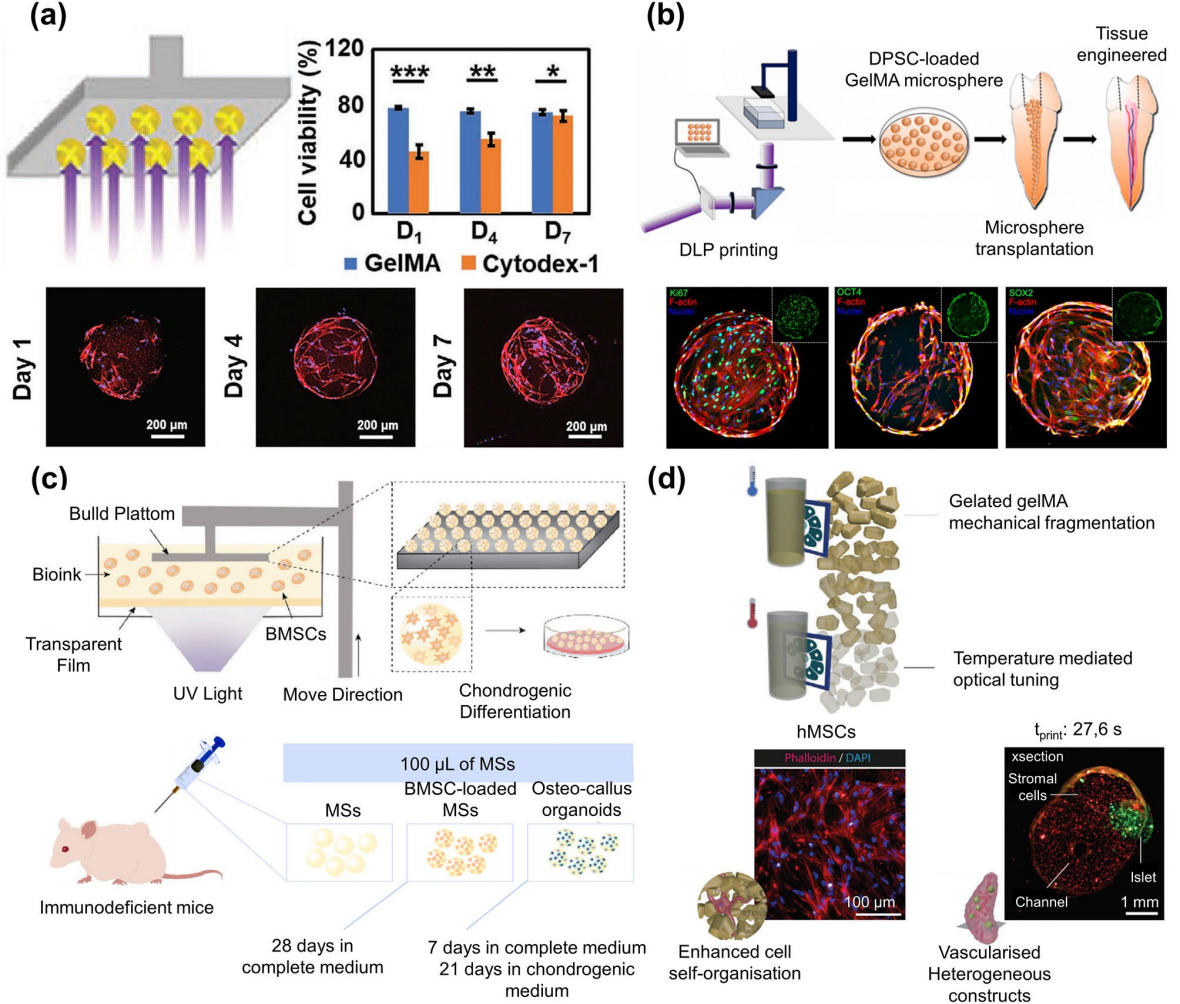
3D bioprinting strategy for preparing microgels. **a** Microgels fabricated via DLP printing for cell culture. Reproduced with permission [99]. Copyright 2020, Wiley–VCH Verlag GmbH. **b** DLP printing fabricated hDPSC-loaded microgels exhibit excellent biocompatibility. Reproduced with permission [100]. Copyright 2023, Elsevier Science Ltd. **c** Bone marrow-derived stem cell (BMSC)-loaded microgels achieved cultivation of osteo-callus organoids for rapid bone regeneration within one month. Reproduced with permission [103]. Copyright 2022, Elsevier Science Ltd. **d** Microgels from embedded extrusion-volumetric printing allowing spatially pattern multiple materials/cell types. Reproduced with permission [17]. Copyright 2023, Wiley–VCH Verlag GmbH

3D bioprinting, as an emerging technology, is combined with the fabrication of microgels. 3D bioprinting technology mainly includes digital light processing (DLP)-based printing [91, 92], inkjet printing [93], extrusion printing [94–96], laser-based printing [97], volumetric bioprinting [98], etc. Hydrogel materials and printing techniques could be selected according to the types of cells and the needs of the growing environment. In addition, different from most other strategies, 3D bioprinting can directly form microgels with complex structures. Same as other methods, this technology is suitable for preparing cell-laden microgels for cell culture. As shown in Fig. 10a, a microgel system integrating cell adhesion, culture, proliferation, collection, cryopreservation and tissue engineering was successfully constructed [99]. Furthermore, human dental pulp stem cells (hDPSCs)-loaded microgels were fabricated via layer-by-layer photocuring using DLP-based printing (Fig. 10b) [100]. These microgels improve angiogenesis and neurogenesis, as well as effectively promote dental pulp regeneration. Traditional cultured organoids based on Matrigel have always faced the challenge of size non-uniformity and take a long time (up to months) [101]. To address this problem, 3D bioprinting has been proposed by many researchers for the cultivation of organoids, since microgels of uniform size can be easily produced in batches by it [102]. For example, about 7000 microgels containing BMSC could be produced at once via DLP-based printing (Fig. 10c) [103]. These microgels can mimic the formation of an osteo-callus during endochondral ossification and subsequently differentiate into bone callus-like structures within only 21 days.

Volumetric bioprinting (VBP), a new 3D bioprinting technology, has been introduced recently [104, 105]. It overcomes the conventional layer-wise bioprinting approaches and the limitation of geometric structure. Printing in situ and obviously improving the printing speed are the most significant advantages of VBP. It can print hydrogels with low mechanical properties in seconds to tens of seconds [106, 107]. This technique is being widely used in the construction of complex tissues and the cultivation of organoids [108]. The combination of VBP with embedded extrusion bioprinting could further improve the efficiency of microgel preparation. In addition, multi-material/complex multi-cellular morphologies could be printed easily (Fig. 10d) [17].

Other microgel fabrication strategies could also be integrated with 3D bioprinting, such as microfluidic technique [109] and gas assistance [110]. With the aid of microfluidic channels and gas, microgels with complex structures including spherical helix, rose, or saddle could be produced [109]. In general, 3D bioprinting is endowed with more possibilities by integrating with other methods.

Although 3D bioprinting still faces many challenges, it is developing at a fast pace and has become a powerful means of printing microstructure and macro-scale organizations. This technology has been utilized for fabricating complex models of hearts, brain tissue, blood vessels, etc. [111]. Despite being in the nascent stages of research, 3D bioprinting exhibits tremendous potential. Its applications across diverse fields, including the development of organoids and fabricating soft tissues, offer exciting and promising prospects for the future.

### Others

In addition to the strategies mentioned above, many other techniques could be used to fabricate microgels, including supercritical fluid, superhydrophobic surface, and supramolecular self-assembly. Microgels prepared by supercritical fluid have the advantages of high monodispersity and roundness [112]. However, since it requires organic solvents, resulting in low biocompatibility, it has been rarely used. The superhydrophobic surface-based strategy provides a universal platform for customizing microgels with special functions and structures [113, 114]. Supramolecular self-assembly refers to the combination of two or more molecules by intermolecular interaction to form complex and highly ordered aggregates [115–119]. Small-size microgels could be generated by supramolecular self-assembly. DNA is one molecule that favors self-assembly into nanostructures. Zeng et al. achieved the preparation of microgels only via homopolymerization of single-stranded DNA motifs, and the size of microgels was 1–5 μm [120]. This strategy did not require multiple unique DNA strands and was simple, and straightforward to implement. However, most of the materials currently used are DNA and polypeptides. Some new materials still need to be developed for self-assembly.

## Microgels for Cell Loading and Delivery

The main purpose of cell delivery is to replace or repair damaged cells and promote the regeneration and repair of tissues or organs. In general, cells are implanted into the damaged site by injection, but due to the fragility of cells, the retention rate and survival rate of cells are mostly low, which may hinder the effectiveness of cell therapy. Microgels can provide protective effect for cells. The cell transplantation rate can increase significantly after encapsulated in microgels. For example, SA microgels have long-term immune protection on islet cells [121]. After implantation of the SA microgels containing islet cells, the foreign-body response was reduced, and insulin deficiency was rectified.

Previous studies have shown that cells would have stronger cell stemness, proliferation, and migration abilities when cultured in microgels because 3D culture microenvironment has better cell–cell and cell-extracellular matrix interactions. Cells in microgels could also be used to build models in vitro, which are widely used in mechanism studies and drug screening [122]. Adjusting the surface curvature of microgels could accelerate cell growth and differentiation. Jin et al. demonstrated that precisely controlling the surface curvature of microgels was beneficial to bone marrow mesenchymal stem cells (BMSCs) growth and osteogenic differentiation [123].

Owing to their high controllability, specific morphology, and variable sizes, microgels have been widely used as excellent carriers for drug and cell delivery to treat different diseases. For the COVID-19 treatment, where the biodistribution of microgels in the lung depends on their diameter, the size of microgels needed to be precisely controlled to achieve an appropriate deposition in the target location [124]. In addition, cells encapsulated in microgels need to carry out effective transport of nutrients and metabolites. The porous controllability of microgels enables cells to continuously absorb nutrients and discharge metabolites in time, providing a suitable microenvironment for cell growth, proliferation, and function. When used for drug release, microgels with adjustable porosity can effectively control the drug release rate, resulting in lower biotoxicity and achieving more effective therapeutic outcomes.

A wide variety of polymers could be used to prepare drug-laden microgels including chitosan, hyaluronic acid, gelatin methacryloyl (GelMA), poly [lactic-*co*-(glycolic acid)], cellulose acetate phthalate, etc. Microgels with high or low stiffness can be prepared by changing the molecular weight and concentration of biopolymers. One critical attribute of microgels is their injectability, which affords them an advantage over traditional open surgery. Through minimally invasive injection, microgels can be delivered into the body, enhancing patient comfort and safety, while simultaneously reducing potential side effects associated with more invasive treatment methods. Furthermore, the efficiency of treatment could be impacted by the morphology of microgels. In this regard, it has been proved that spherical microgels have better injection performance and self-healing ability compared to irregular microgels prepared through mechanical crushing [125]. The immune cells and islet cells were co-injected into the gap of the microgels. Due to their lower extrusion pressure of injection, the spherical microgels demonstrate more evenly distributed cells and stronger cell vitality.

There are several strategies to incorporate cells in microgels; cell encapsulation in microgels, cell adsorption on microgels, and single-cell encapsulation. The relative position between microgels and cells as well as the number of cells could affect the growth and differentiation of cells. In this section, different packaging methods, including multicellular and single-cell encapsulation, and their applications in various fields are introduced. Also, the differences in the applications of these packaging methods are comprehensively discussed as a guideline for future research directions.

### Microgels for Cells Delivery

**Fig. 11:**
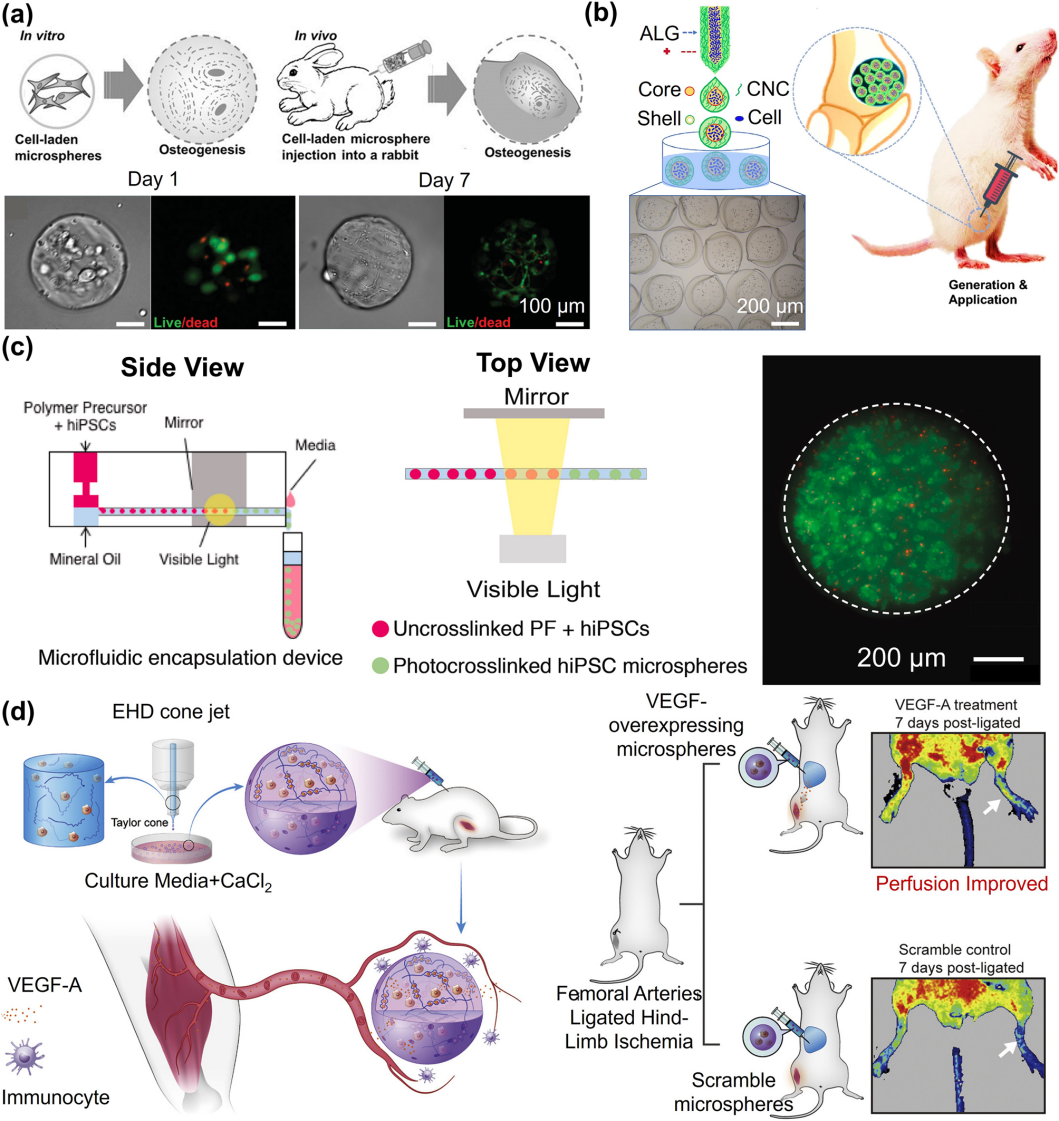
Cell culture and delivery in microgels. **a** Microgels provide immune protection to islet cells, improve cell transplantation rate and correct insulin deficiency. The cell viability remained highly 7 days after encapsulation. Reproduced with permission [128]. Copyright 2016, Wiley–VCH Verlag GmbH. **b** Hydrogel shell containing cellulose nanocrystal (CNC) increases the mechanical strength and provides better protection in treating bone defects. Reproduced with permission [129]. Copyright 2022, Shanghai Jiao Tong University Press. **c** hiPSCs wrapped in PEG-fibrinogen microgels differentiated into self-contracting engineered heart tissue. Reproduced with permission [131]. Copyright 2021, Elsevier Science Ltd. **d** Microgels encapsulating vascular endothelial growth factor-overexpressing HEK293T cells for inducing angiogenesis in limb ischemia [132]. Copyright 2020, Elsevier Science Ltd

Microgels, as highly biocompatible materials with biological functions, have been widely used in cell culture [126, 127]. Single or multiple cells can be encapsulated in microgels via appropriate fabrication methods. Beyond working as cell delivery carriers, microgels can create a 3D controllable microenvironment conducive to cell proliferation and distribution. Recently, cell-laden microgels have made some progress in the field of bone tissue engineering. Zhao et al. reported a strategy for bone tissue repair by encapsulating BMSCs and growth factors in GelMA microgels and using them as osteogenic constructs (Fig. 11a) [128]. Owing to the protective effect of the microgels on the cells, the survival of the cells was prolonged, and the differentiation of cells into functional osteoblasts was more favorable. An all-aqueous-phase microfluidic electrospray system was developed to form a novel stem cell delivery microgel system with a biomass shell made of cellulose nanocrystal (CNC) and alginate (Fig. 11b) [129]. The absence of oil and surfactant and the presence of CNCs gave the microgels greater mechanical strength and a porous structure, which allowed adequate material exchange between the cell and the environment. These microgels showed satisfactory biocompatibility and acceptable therapeutic effects in the subsequent bone defect models.

Since many native tissues, such as osteochondral tissue, are made of multiple types of cells, only one type of microgel may not be beneficial for tissue repair. Cui et al. proposed a combinatory treatment method using two types of microgels [130]. Thiolated heparin (HS) and strontium nanoparticles (SNPs) were integrated into GelMA and subsequently co-encapsulated with equine umbilical cord blood-derived MSCs to prepare two types of microgels. These microgels formed engineered osteochondral cartilage tissue and osteoblast layer with the aid of 3D printing technology. Specifically, SNPs have been shown to prompt osteoblast differentiation of cells, whereas HS has been conducive to the development of hyaline-like cartilage. Thus, under a combined action, microgels could promote the regeneration of the osteochondral tissue. As shown by another study, MSCs and human umbilical vein endothelial cells (HUVECs) could be also separately encapsulated in two independent compartments of Janus microgels [56]. The interaction between the two cell types affected the function of stem cells and promoted osteogenic differentiation.

Since cardiomyocytes are nonregenerative, the development of cardiomyocyte therapy has been hindered to a large extent. Alternatively, human-induced pluripotent stem cells (hiPSCs) were encapsulated in poly (ethylene glycol) (PEG)-fibrinogen microgels using a modified microfluidic oil-and-water emulsion technique [131]. The encapsulated hiPSCs were able to differentiate into self-contracting engineered heart tissue (Fig. 11c). Engineered heart tissue derived from hiPSC-laden microgels could spontaneously contract and respond to drug therapy and electrical stimulation. Similarly, human embryonic stem cells (hESCs) were encapsulated in collagen-type I microgels for the hepatic differentiation. The hESCs in microgels were efficiently differentiating into hepatocyte-like cells (HLCs). These HLC-loaded microgels constructed prevascularized liver tissue (PLT) via self-assembly with endothelial cells. After PLT was implanted in mice, significant improvement was observed in the liver function [122]. Apart from bone and cardiac muscle repair, cell-laden microgels are also widely used in angiogenesis [132] (Fig. 11d) and hair regeneration [133]. Microgels coated with HEK293T cells that overexpressing VEGFA could significantly improve blood flow in injured hind limbs. Core–shell microgels containing growth factors and two types of cells could support cell proliferation and release growth factors continuously, thus efficiently producing hair follicles.

**Fig. 12:**
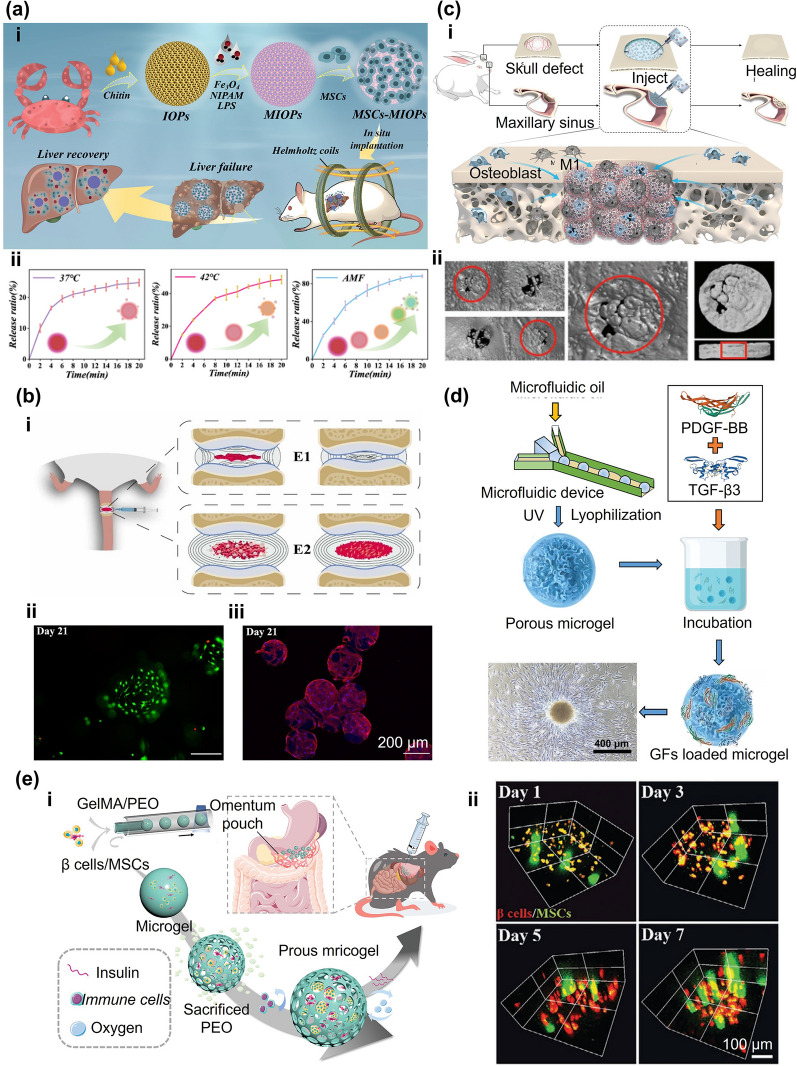
Cell culture and delivery on microgels surface. **a** (i) MSCs were loaded onto the surface of MIOPs and applied to the treatment of ALF. (ii) LPS release profiles of the MIOPs treated with heating and alternating magnetic field (AFM). Reproduced with permission [134]. Copyright 2022, Wiley. **b** (i) GelMA microgels enhanced the in vitro differentiation of rASD into NP-like cells and partially restored the degenerated intervertebral disc. LIVE/DEAD staining (ii) and TRITC-phalloidin staining **(**iii**)** of microgels after 21 days. Reproduced with permission [135]. Copyright 2021, Iop Publishing Ltd. **c** (i) IL-4-loaded microgels stimulate cells to differentiate into M2 type. Reproduced with permission. (ii) Representative Micro-CT image of rabbit calvaria healing 8 weeks after injection [137]. Copyright 2022, Wiley–VCH Verlag GmbH. **d** PDGF-BB recruits stem cells to adsorb on microgels and differentiate into cartilage. Reproduced with permission [138]. Copyright 2022, Wiley–VCH Verlag GmbH. **e** (i) β cells and mesenchymal stem cells were co-encapsulated in porous microgels for controlling type I diabetes. (ii) 3D reconstructed images of the β cells (red) and MSCs (green) inside the porous microgel at 1, 3, 5, and 7 days. Reproduced with permission [139]. Copyright 2023, Wiley–VCH Verlag GmbH

In the one-step method, although cell encapsulation in microgels has high cell-loading density, cell loading and preparation of microgels, are often carried out simultaneously. Therefore, the preparation method and subsequent processes, such as the mechanical stress on the cells during the process of mixing into the hydrogel solution and preparing into microgels, may affect cell viability. The two-step approach, in which the microgel is prepared first and then attached cells to the surface of microgels could be a solution to this problem. Compared to encapsulated cells inside the microgels, the cells on the surface are not restricted by the mechanical forces within the hydrogel network. As a result, the seeded cells maintain high viability and migration rates, and they can be collected and separated conveniently. This method allows for cell loading after microgel fabrication, mitigating cellular damage from crosslinking, oil extraction, or related procedures and providing greater latitude in choosing hydrogel precursors. This method gives more flexibility in selecting hydrogel precursor solutions. For example, MSCs could be loaded onto the surface of novel lipopolysaccharide (LPS)-composited magnetic-thermal responsive inverse opal microgels for the treatment of acute liver failure (ALF) (Fig. 12a) [134]. The microgels were filled with poly(N-isopropylacrylamide) (PNIPAM) hydrogel containing LPS and Fe_3_O_4_ nanoparticles. The magnetothermal conversion characteristics of Fe_3_O_4_ and the thermal response behavior of PNIPAM provided the microgels with magnetothermal responsiveness. LPS could be released under the stimulation of an alternating magnetic field, thus activating MSCs to obtain the characteristics of the “trained immunity”. Structural color changes of the microgels could also monitor the process. Considering such excellent properties, the implantation of MSC-adsorbed microgels in rats with ALF showed significant anti-inflammatory and therapeutic effects.

In tissue repair, cells play a leading role in treatment. Growth factors and substances, such as exogenous cytokine, help cell growth, differentiation, tissue repair, and achieving better therapeutic outcomes. Xu et al. fabricated GelMA microgels and loaded stem cells and growth factors via the electrospray method (Fig. 12b) [135]. Based on the good biocompatibility and mechanical properties of GelMA, the microgels served as delivery carriers for cells and growth factors. Employing growth factor-loaded microgels also enhanced the differentiation of embedded cells into nucleus pulposus-like phenotypes, which could partially restore the degenerated intervertebral disc. Similarly, IL-4-loaded liposomes were chemically modified on poly (L-lactic acid) microgels through amide bonds (Fig. 12c) [136]. When cells were adsorbed on the microgels, they could transmit the molecular signal to macrophages and stimulate them to differentiate into M2 type. Therefore, osteoblast proliferation and differentiation were promoted and bone regeneration was accelerated.

In addition to directly loading or attaching cells to the microgels, specific growth factors or antibodies can induce cells to aggregate and attach to the microgels. Inspired by the nesting phenomenon of seabirds, Lei et al. integrated growth factors into methacrylate hyaluronic acid (HAMA) and heparin blend microgels through the microfluidic strategy [137]. Their method increased cell migration ability, released platelet-derived growth factor-BB to recruit stem cells, and eventually induced the recruited cells to differentiate into cartilage by releasing transforming growth factor-β-3, which was beneficial to the treatment of osteoarthritis (Fig. 12d). However, the recruitment of BMSCs using a single marker is not always straightforward for in situ bone regeneration. Sun et al. prepared CD271 functionalized microgels that can capture BMSCs from the surrounding environment under the action of CD271 [138]. Because of the polydopamine coating on the microgels, the trapped cells were provided with extremely good conditions for stretching and proliferating on the surface of the microgels. Implanted microgels significantly promoted new bone formation in the femoral condylar defect area, providing a suitable vehicle for bone regeneration in situ.

It should be noted that microgels with low porosity may not provide sufficient internal space for cell proliferation. The proliferation and migration of cells will be limited for some hydrogels with high mechanical strength and low degradation rates, such as SA [56]. Besides, the pressure and friction between the microgels can induce apoptosis during injection. Although cell adsorption on the surface of the microgels is helpful for cell elongation and proliferation, it eliminates the protective effect of the microgels on the cells. Porous microgels are great options for both protecting cells and meeting the needs of cell extension and growth. Previous studies have used porous microgels in treating type I diabetes, also known as insulin dependence [139]. The need for continuous administration of exogenous insulin due to the destruction of islet β cells and the absolute insufficiency of insulin secretion spurred the researchers to investigate porous microgels for this prevalent disease. Specifically, β cells and MSCs were co-encapsulated, and then porous microgels were obtained by sacrificing polyethylene oxide (PEO) (Fig. 12e). Porous structures of microgels promoted the growth of β cells and improved their insulin secretion function. This was conducive to achieving sustained glycemic control. Furthermore, the porosity could be controlled by changing the ratio of PEO to GelMA. The microgels with good porosity and mechanical properties ensured the supply of nutrients and oxygen to the cells, and excellent permeability for the generated insulin. Similarly, BMSC cells were encapsulated in porous microgels prepared from alginate to protect them from immune cell attack while maintaining their immunomodulatory function for the treatment of systemic lupus erythematosus (SLE) [140]. Alginate formed porous microgels by immediate gelling reaction with poly-D-lysine (PDL). Thanks to the electrostatic adsorption and covalent bonding between PDL and the tissue, the porous microgels could adhere tightly to the intestinal surface after intraperitoneal injection, which could transform the activated inflammatory macrophages into anti-inflammatory states and effectively improve the treatment course of SLE. In addition, cells can attach to the surface of the porous microgels similar to microgels with low porosity. Human periodontal ligament stem cells could be implanted on silk fibroin and/or hydroxyapatite-modified PLGA microgels through a special cell perfusion technique [141]. Due to the excellent cell adhesion of fibroin, microgels showed excellent tissue repair effects in periodontal tissue regeneration.

In addition to delivering cells, microgels can be used to transport liposomes [142], proteins [143], exosomes [144], and growth factors [145]. When they are used to pack stimulus-responsive substances, these microgels' movements can be precisely controlled, enabling targeted delivery of drugs and cells and effectively acting as microrobots [3, 33, 146]. Consequently, microgels are excellent carriers for the delivery and treatment of cells, bioactive substances, and drugs [147, 148].

### Single-Cell Encapsulation in Microgels

**Fig. 13:**
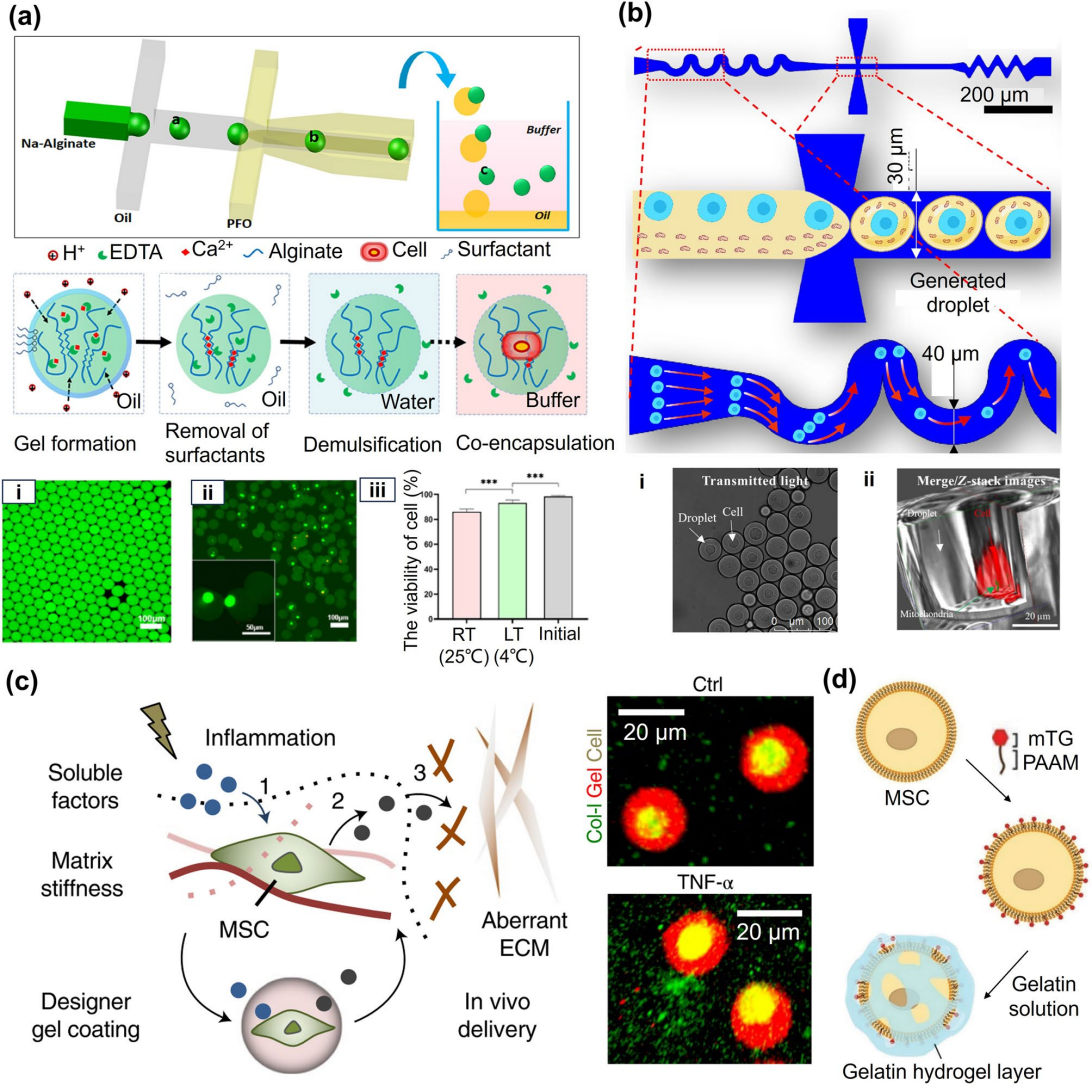
Single-cell encapsulations in microgels. **a** Encapsulating single mesenchymal stem cells in alginate microgel through microfluidic. Reproduced with permission [149]. Copyright 2020, Elsevier Sci Ltd. **b** Increase the wave-like structure and improve the single-cell encapsulation efficiency to achieve high-efficiency quantitative control of mitochondrial transfer. Reproduced with permission [150]. Copyright 2022, American Association for the Advancement of Science. **c** MSCs encapsulated in SA gel for facilitating the reversal of aberrant tissue remodeling. Reproduced with permission [23]. Copyright 2022, Nature Research. **d** Fabrication of single-cell microgels by chemical modification. Reproduced with permission [152]. Copyright 2021, Wiley

Current cell encapsulation technologies are advancing feasibility in tissue engineering and regenerative medicine such as cell therapy, and in vitro models. Recent studies have shown that microgels can be miniaturized to the single-cell scale, where only one cell is packed in a microgel. Unlike the traditional multicellular packaging, single-cell encapsulation separates individual cells, resulting in a larger ratio of surface area to volume for the cells. Smaller microgels are adequate when single-cell encapsulation is used. The thinner gel layer improves the exchange rate of nutrients and metabolic waste, avoids central necrosis, reduces immune response, and improves cell survival rate after transplantation (Fig. 13a) [149]. However, there are intrinsic challenges associated with single-cell encapsulation. Traditional microfluidic devices with low single-cell encapsulation efficiency are mostly compatible with cell-free microgels or multi-cell encapsulated microgels. Although the single-cell encapsulation efficiency could be increased to 47.8% by modifying the microfluidic devices by adding wave-like structures, the effect was still not ideal (Fig. 13b) [150].

Researchers have adopted various optimization schemes to prepare high-quality single-cell microgels. For example, microfluidic electrospray has been modified to work as a high-throughput single-cell coating method [77]. With the tip voltage set to alternating current (AC) and using a sharp conical semilunar surface, only one cell ejected at a time which improves encapsulation efficiencies over by 80%. Unlike using direct current for encapsulating multiple cells in a droplet, AC avoided the recycling in the Taylor cone, streamlining the generation of single-cell encapsulated microgels. The method was compatible with different natural and synthetic materials. Using this method, by encapsulating single human mesenchymal stem cells in microgels with an average wall thickness of about 4 μm, the cells maintained more than 80% viability and preserved their robust osteogenic or adipogenic differentiation potential.

Cell surface modification, which is divided into physical and chemical modifications, has received extensive attention to further improve the efficacy of cell therapy. Due to its acceptable biocompatibility and fast crosslinking properties, SA is used in single-cell encapsulation research in the early stages. In a previous study, the cells were incubated with Ca_2_O_3_ particles so that some particles attach to the cell surface, and then they were embedded in SA [151]. Because of the rapid gel reaction between Ca^2+^ and SA, the cell surface was coated with a gel layer. The thickness and mechanical properties of the gel were determined by the number of particles and the polymer's molecular weight. The gel thickness surrounding the cells could reach to values as small as 10 μm. The encapsulated MSCs not only secreted twice as much IF-6 compared to unencapsulated cells but also in response to the presence of TNF-α secreted soluble interstitial collagenase, which could reverse abnormal tissue remodeling (Fig. 13c) [23].

In addition to physical adsorption, cell surfaces can also be modified by chemical reactions. For this purpose, researchers employed microbial transglutaminase (mTG), which could catalyze gelatin crosslinking (Fig. 13d) [152]. Polysialic acid anchor for cell membranes (PAAM) was linked to mTG via N-hydroxysuccinimide to form the PAAM-mTG molecule. Then the PAAM was inserted into the lipid bilayer of the cell so that mTG was exposed to the outer surface of the cells. With the coupling of gelatin molecules, the cell surface could be coated with a nano-gel layer with a thickness of 332.0 ± 85.8 nm. The coating did not significantly change the functional activity of the cells. MSCs with nano-gel layer remarkably restored cardiac function and reduced myocardial infarction size. The original groups on the cell surface can also be modified for single-cell encapsulation. For example, the disulfide in the cell surface protein could be gently reduced by tris (2-carboxyethyl) phosphine, followed by mercaptan-maleimide coupling, which was suitable for a variety of cell encapsulation processes [153]. When the encapsulated single cells were implanted, they could be controllably released at specific target sites. For example, high expression of human matrix metalloproteinase-7 at the tumor site could break peptide chains in the PEG-gelatin layer and release encapsulated cells in situ [154]. Overall, single-cell encapsulation improves flexibility and further facilitates the application of cell-carrying microgels in biomedical applications.

## Microgels-Based Scaffolds for Tissue Engineering and Regenerative Medicine

The fields of tissue engineering and regenerative medicine aim at repairing tissue damage through applying cell-laden or acellular bulk hydrogels. However, the nano porosity of available hydrogels has imposed limitations in recruiting surrounding cells or transferring signaling cues and nutrients to encapsulated cells. Even when bulk hydrogels with micro-scale porous structures are employed to facilitate the transport of cells, signaling, and nutrients, the translocation and heterogeneous regeneration for a large volume remains limited. In addition to serving as a granular cell carrier, microgel assembly offers multistage structural microporous scaffolds for promoting tissue regeneration and microtissue assembly by providing both nano-porosity from the material and microporosity from the structural assembly [155, 156]. Wei et al. developed a highly porous injectable carrier, polyhydroxyalkanoate open porous microgels (PHAOPMS), via combining the advantages of microgels and scaffolds, providing enough open 3D space for cell attachment, proliferation, and migration [157]. This system protected cells from stress during injection, while safely transporting them to the defect area. BMSCs transported with PHAOPMS possessed stronger osteoblast regeneration capability and were able to successfully remodel bone tissue subcutaneously, accompanied by more vascularization. In addition, microgel scaffolds were used to regulate local excessive inflammation to inhibit intervertebral disc degeneration and promote its regeneration [158].

Apart from cell therapy, microporous scaffolds are also an effective way to establish tumor models and culture tumor cells for assessing their original tumor stemness, proliferation, migration ability, and drug resistance. He et al. used honeycomb-like porous GelMA microgel scaffolds to culture osteosarcoma cells (K7M2) [24]. The 3D cultured K7M2 cells showed stronger tumorigenicity, which was characterized by shorter tumor formation time, larger tumor volume, severe bone destruction, and higher mortality. The microgel system provided the researchers with an effective and convenient microenvironment for tumor pathogenesis and drug screening assays. Based on the special structure of the scaffold, it could promote vascular regeneration and bone formation even in the case of acellular bone formation [27].

Although using a single microgel as a scaffold has great advantages, they are easy to flow and difficult to maintain their morphology when introduced in large defect areas. Therefore, it is difficult to achieve the purpose of treatment. The limitation of single-microgel scaffolds lays a foundation for the development of scaffolds composed of multiple microgels. According to recent studies, scaffolds made of multiple microgels can be fabricated via many techniques. In the following, these techniques are divided into two categories depending on whether the particles form into a scaffold through external or internal forces.

### Scaffolds Formed by the Interforce

**Fig. 14:**
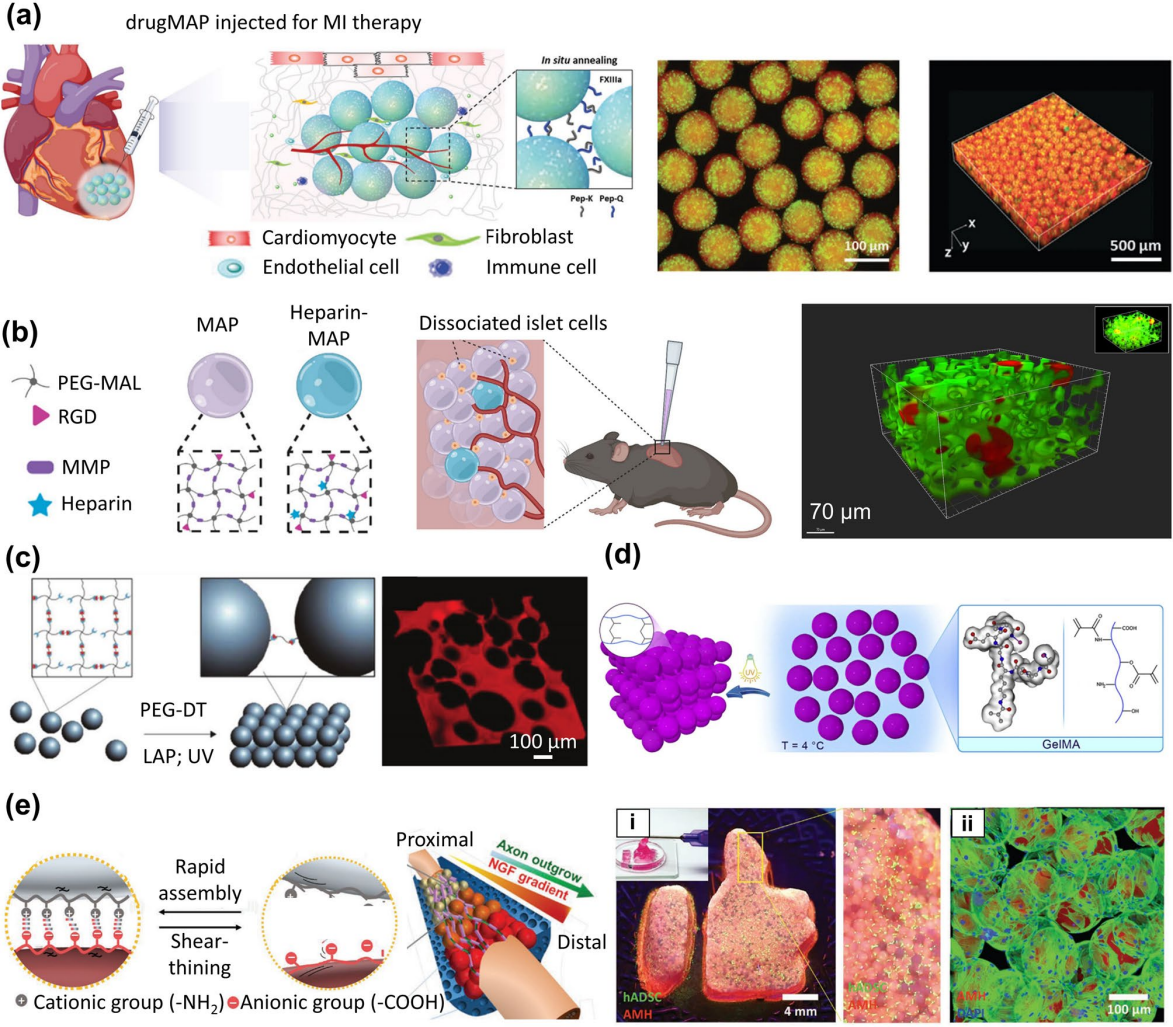
Microgel scaffolds formed by interforces. **a** MAP scaffold for myocardial infarction drug delivery. Reproduced with permission [162]. Copyright 2020, Wiley–VCH Verlag GmbH. **b** MAP scaffolds as delivery carriers of islet cells were applied in type I diabetes. Reproduced with permission [166]. Copyright 2022, Springer Healthcare. **c** The annealing degree of the overall scaffold was directly controlled by changing the ratio of tetrazine to norbornene. Reproduced with permission [167]. Copyright 2018, Wiley. **d** Using the temperature sensitivity of GelMA, scaffolds are formed by physical crosslinking. Reproduced with permission [169]. Copyright 2019, Elsevier Science Ltd. **e** GelMA and ChitoMA microgels form scaffolds through electrostatic interaction. (i) Injectable and moldable hydrogel formed complex and macroscale morphologies. (ii) Fluorescence image of the microgel-based scaffold. Reproduced with permission [170]. Copyright 2019, Wiley

Microporous annealed microgels (MAP), an injectable biomaterial composed entirely of microgels, have been widely used in the construction of scaffolds due to their excellent injectability and in situ annealing properties. Lattices of MAP microgels, annealed with each other by surface functionalities, can be injected and molded into any morphology, providing mechanically stable scaffolds for cell growth with an interconnected network of micron-scale micropores. Compared with the direct delivery of stem cells and nanoporous hydrogels, the micron-scale pores of MAP scaffolds enhance the transport of nutrients, promote cell migration and intercellular connections, and increase the retention of cells in the subcutaneous area [159, 160]. MAP scaffolds had been previously used to accelerate skin wound healing [161]. Recently, their use in other regenerative medicine and tissue engineering applications has been successful, and they could serve as drug delivery platforms to reduce fibrosis and treat myocardial infarction (Fig. 14a) [162]. Replacing or rebooting tissues or organs damaged due to disease, injury, age, or other issues is a major goal of regenerative medicine, which entails a balance between scaffold degradation and tissue regeneration. In this regard, switching the chirality of MAP crosslinked peptides from L- to D-amino acids was attempted in wound healing [163]. The results showed that the MAP scaffolds simultaneously accelerated the degradation rate in vivo and induced skin healing without the assistance of stem cells, growth factors, or adjuvants.

MAP scaffolds are also widely used in diabetes treatment. Diabetic wound healing has not been satisfactorily treated due to issues such as hyperglycemia, hypoxia, increased expression of pro-inflammatory cytokines, and bacterial infection [164]. Recently, Pruett et al. proposed heparin microislands (μIslands), in which heparin-containing microgels were incorporated into microporous annealed microgel scaffolds, to organize endogenous growth factors and significantly improve wound healing in the diabetic wound environment [165]. Based on its epidermal regeneration and revascularization capabilities, MAP scaffolds, as delivery carriers of islet cells, were shown to be beneficial in type I diabetes (T1D). The results showed that the scaffold retained the function of glucose sensing and insulin secretion, and maintained the normal blood glucose of the type I diabetes model. This approach was expected to solve the problem of insufficient donors of pancreas or islet transplantation for patients with T1D (Fig. 14b) [166]. Despite this excellent progress, MAP scaffolds still lack adjustability in stiffness, making them difficult to meet the needs of various applications. Thus, to address this critical issue, microgels were first synthesized via the thiol-norbornene click reaction, and then annealed into porous scaffolds using the tetrazine-norbornene click reaction (Fig. 14c) [167]. This assembly method allowed the annealing degree of the overall scaffold to be directly controlled by changing the ratio of tetrazine to norbornene, providing suitable adhesion sites for the cells. MAP scaffolds with lower annealing degrees allowed cells to grow rapidly and in large quantities, which may be attributed to the cells' ability to move freely in the less crosslinked and less stiff scaffolds [167, 168]. Compared with the use of extra functional groups for annealing, GelMA can directly prepare annealing-controllable scaffolds due to its unique temperature sensitivity. For example, GelMA microgels were made to have fluidity by temperature change and were physically crosslinked to form scaffolds (Fig. 14d) [169]. Then, GelMA scaffolds with high mechanical properties were prepared by photochemical annealing. Scaffolds prepared by this strategy had more controllable porosity and stiffness, providing a favorable microenvironment for cell growth. Highly elastic, self-healing microgel-based scaffolds can be assembled by controlling microgels with opposite electrical charges of the hydrogel. Unlike MAP scaffolds that undergo chemical crosslinking, physical crosslinking can reduce the residual chemicals and increase biocompatibility. For instance, the treatment of peripheral nerve injury has always faced the problem of weak growth factor propagation gradients. GelMA and chitosan methacryloyl (ChitoMA) microgels, which could be photocrosslinked and have negative and positive charges respectively, were interconnected to form adjustable and interconnected porous scaffolds for the treatment of peripheral nerve injury (Fig. 14e) [170]. Simultaneous loading of gradient concentration of nerve growth factor (NGF) into microgels, combined with the gradient propagation of NGF and porous channels, effectively promoted the migration of Schwann cells, induced bridging effects at nerve injury sites, and enhanced axonal outgrowth. Injectable scaffolds with self-assembly and self-editing properties can fill complex morphology defects in skin wound healing. For instance, when BP-contained ChitoMA with the infrared response and bFGF-contained HAMA microgels were injected into the defect, the electrostatic interaction between positively charged ChitoMA and negatively charged HA induced the microgels to spontaneously form scaffolds [171]. Combined with infrared irradiation, scaffolds exhibited great antibacterial activity and promote macrophage proliferation, migration, and M2 polarization. Furthermore, changes in the microenvironment, such as reduced inflammatory response, promoted neovascularization and collagen deposition, further accelerating the wound healing process.

### Scaffolds Formed by the External Force

In tissue engineering and regenerative medicine, porosity is a key therapeutic factor for microgel scaffolds. Porosity is crucial in transport efficiency of oxygen and nutrients, diffusion of therapeutic factors, thus directly affects the proliferation and spread of cells in or outside scaffolds. When the external force is applied, greater control over the range of porosity and the morphology complexity of scaffolds may be achieved, comparing to scaffolds formed solely based on the interactions between microgels. Generally, the external force assistance can be divided into two categories. The first category is the sacrificial microgel scaffold prepared by the template method. In this approach, another phase is added to fill the gap between the microgel, and then the microgels are removed by physical or chemical means to form a scaffold with a defined pore structure [25, 172–176]. The second category is 3D printing microgel scaffold, in which macroscopic scaffolds with or without the presence of the second phase is constructed [177–180].

**Fig. 15:**
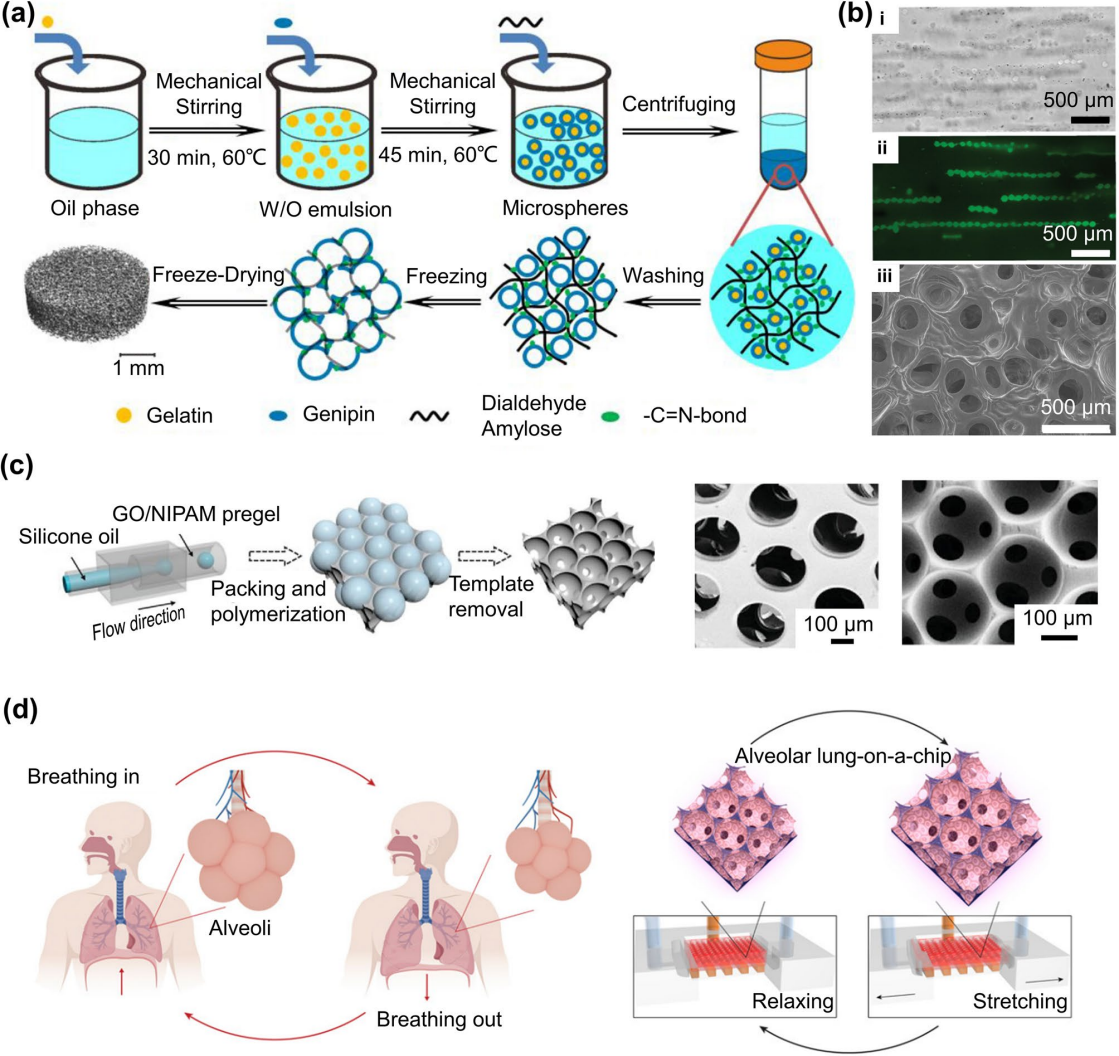
Microgel scaffolds formed by external forces. **a** Gelatin-based scaffolds with controllable pore structures were prepared by the emulsion template method. Reproduced with permission [174]. Copyright 2021, American Chemical Society. **b** Combined with an external magnetic field, the microgels are assembled into a specific pattern, which is conducive to nerve repair. (i) Light microscopy image of the magnetically templated hydrogel. (ii) Confocal microscopy image of FITC-dextran backfilled templated hydrogel. (iii) An SEM image of a magnetically templated hydrogel. Reproduced with permission [182]. Copyright 2019, Academic Press Inc. **c** HepG_2_, 3T3, and HUVECs, were inhaled into scaffolds and co-cultured to form a liver-on-a-chip system. Reproduced with permission [172]. Copyright 2019, American Association for the Advancement of Science. **d** Scaffold as a 3D tissue model for biological exploration and in vitro drug test. Reproduced with permission [25]. Copyright 2021, Proceedings of the National Academy of Sciences of the United States of America

An improved emulsion template method was reported for fabricating gelatin-based scaffolds with controllable pore structures (Fig. 15a) [174]. Genipin was added to the gelatin microgels to solidify the outer layer of them. A biomacromolecule crosslinking agent, dialdehyde amylose, was then added into the emulsion to form a scaffold from the microgels. The unreacted gelatin in the center was washed with water, and the porous scaffold was obtained by freeze-drying. The pore size could be easily controlled by changing the size of gelatin microgels. This biocompatible material system is also suitable for the fabrication of drug-carrying porous scaffolds. The porous structure of scaffolds combined with the mechanical properties conferred by SA made the scaffolds sufficiently compressible to enter the uterus through the vagina [181]. The drug was released gradually via the interconnected pore structure of scaffolds. The scaffolds could cellularize the damaged tissue and repair the endometrium. It was proved that these scaffolds were suitable for the intrauterine adhesion treatment.

For tissue repair, mimicking of native structure of the target tissue is of critical importance as the functional characteristics of body organs, such as the heart, bone, and tendons are closely related to their unique structure. Specifically, the anisotropic aligned structure of the nerve tissue was achieved using a magnetic field. For this purpose, photocrosslinkable glycidyl methacrylate hyaluronic acid was employed as the external phase, and the SA microgels containing magnetic iron oxide (IO) were removed by EDTA to form a porous scaffold for 3D cell culture (Fig. 15b) [182]. When an external magnetic field was applied, the microgels were assembled into a designed pattern, and with the increase of the concentration of IO, the ability to align with the magnetic field was improved. This unique feature was advantageous for mimicking the neatly arranged and highly anisotropic tissue structure of the native nerve tissue.

In addition to pre-dissolving microgels in vitro, different degradation strategies could be used to preferentially degrade one of the two phases to form a porous structure in vivo. This strategy has been used for making biomimetic 3D tissue models with applications in physiology and pathophysiology [173]. Comparing to 2D tissue models, such 3D models provide more reliable platforms for understanding biology at the tissue level as well as drug testing. Novel photo-controlled shrinkable inverse opal graphene oxide (GO) hydrogel scaffolds have been proposed, inspired by the hunting process of marine predators in nature (Fig. 15c) [172]. Based on the contraction stress generated by the scaffold, three different types of cells, HepG_2_, mouse embryo fibroblast NIH3T3 (3T3), and HUVECs, were inhaled into porous scaffolds and co-cultured to form liver-on-a-chip systems. They achieved dynamic cell culture and effectively tested specific liver functions, including albumin secretion, urea synthesis, and cytochrome P450 expression. In addition, the inverse opal hydrogel structure with clear and interconnecting pores that were highly similar to human alveolar sacs was an ideal material for simulating the structure of alveoli (Fig. 15d) [25]. With the assistance of 3D scaffolds, cell growth, apoptosis, gene expression, and mitochondrial function were more similar to the in vivo environment [183]. More interestingly, the scaffold perfectly simulated the normal breathing state, and the pressure or respiratory frequency on the chip could be controlled by changing the size of the chambers on both sides or the frequency of negative pressure applied.

**Fig. 16:**
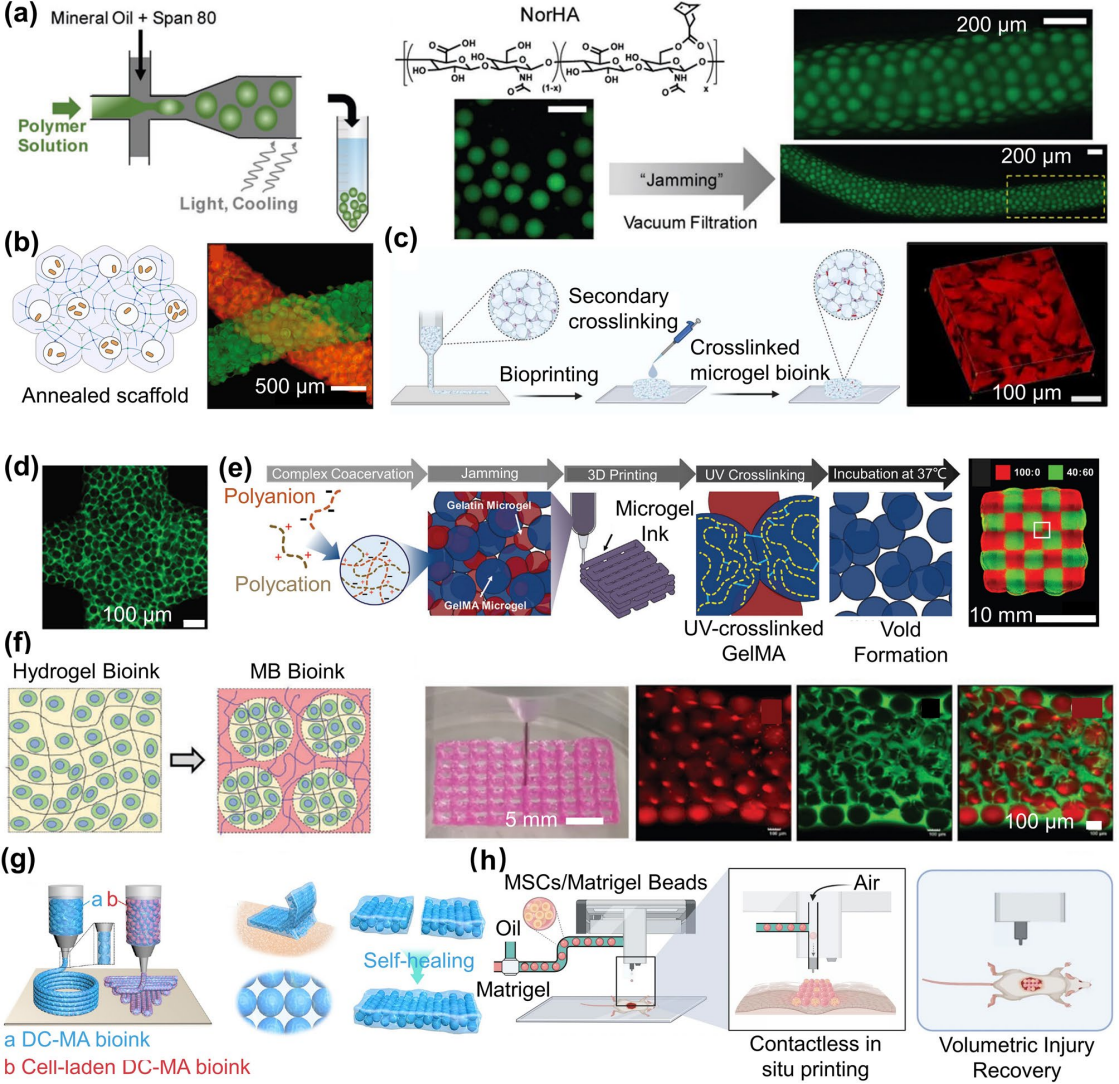
Microgel scaffolds formed by 3D bioprinting. **a** Jammed microgel ink can quickly self-heal and have obvious pores. Reproduced with permission [191]. Copyright 2019, Wiley. **b** Core–shell microgels protect cells and prevent cell leakage during printing. Reproduced with permission [192]. Copyright 2023, Nature Publishing Group. **c** Irregular microgels were used in 3D printing to prepare scaffolds. Reproduced with permission [178]. Copyright 2022, Iop Publishing Ltd. **d** Fabrication of 3D porous scaffolds by sacrificial particle technology. Reproduced with permission [193]. Copyright 2022, Wiley. **e** Two kinds of microgels were mixed, and scaffolds with certain porosity were prepared by sacrificing one of them. Reproduced with permission [180]. Copyright 2021, Wiley. **f** Microgels and the solution together are used as the printing ink. Reproduced with permission [177]. Copyright 2021, Wiley–VCH Verlag GmbH. **g** Strengthening microgel interactions through dynamic covalent bonds guarantees high morphology fidelity and cellular activity. Reproduced with permission [194]. Copyright 2022, American Chemical Society. **h** Bead-jet printing enhances skeletal muscle and hair follicle regeneration with non-uniform cell concentrations. Reproduced with permission [195]. Copyright 2022, Nature Publishing Group

Tissue repair often faces difficulties such as inappropriate scaffold size, complex morphology mismatching, and immune response. Nevertheless, many scaffold characteristics, including pore morphology, porosity, and connectivity between pores, can be accurately adjusted by 3D bioprinting technology [184]. Due to its excellent biocompatibility, degradability, photo-responsiveness, and printability, hydrogels have become a common bioink used in tissue engineering and regenerative medicine [185–189]. However, the scaffold prepared using bulk bioink has large pores, high density, and a small adjustable range, which makes it difficult to meet the treatment needs of various diseases. Alternatively, microgel bioinks, which consist of pure microgels or microgels mixed into another phase have several advantages. Microgels are highly versatile in terms of appropriate biological material selection, size, and morphology according to treatment requirements. Based on the micrometer-scale size of the microgels and the weak physical interactions between them, the bioinks containing microgels with shear-thinning behavior are conveniently extruded out of small-diameter needles [190]. In addition, since the microgels only rely on non-covalent interactions, jammed microgel bioinks can quickly self-heal and have obvious pores for cell migration, proliferation, and differentiation (Fig. 16a) [191]. This injectability as well as porosity gives the particulate bioink excellent macroscopic printability, making it a feasible option for applications in bioengineering. However, the scaffold formed by jammed microgel bioinks has only short-term stability. If long-term stability is to be achieved, a secondary crosslinking agent can be introduced for further fixation. Of note, although the cells can be encapsulated in the microgels and have a satisfactory survival rate, they tend to leak out during the extrusion process and after printing. Hence, it is often necessary to weigh the printability of materials against the adaptability of cell growth. Accordingly, core–shell microgels were used as bioinks to reduce cell leakage to the medium while providing two different microenvironments for cell growth (Fig. 16b) [192]. Different from monolithic microgel scaffolds, core–shell microgel scaffolds showed better spatial separation. Also, the single microbial populations wrapped in the core part enhanced the bioprocessing capability with more significant biological activity. Other studies have shown that scaffolds prepared from novel types of microgels made of ChitoMA and polyvinyl alcohol could form cell spheroids and be applied in the field of organoids and tissue engineering [26].

Irregular microgels have also been employed in 3D printing to fabricate scaffolds. Unlike spherical microgels, the irregular surface causes greater friction between microgels and increases the stability of the scaffold (Fig. 16c) [178]. The size of irregular microgels was determined by the required printing resolution and the target therapeutic needs. Smaller microgels not only proffered higher printing resolution and structural stability but also caused the printed scaffold to induce lower immunogenicity, which can continuously boost tissue maturation and regeneration. Sacrificial microgel technology has been also used in combination with 3D printing technology, where the microgels are employed as the supporting bath (Fig. 16d) [193]. By mixing two kinds of microgels to prepare scaffolds and sacrificing only one of them, porous scaffolds could be fabricated [180]. Changing the mixing ratio of the microgels gave the researchers the capability to further adjust the porosity and enhance cell mobility. Since the microgels were sacrificed after printing the scaffold, this strategy decoupled the printability of the microgel bioinks from the porosity of the scaffolds (Fig. 16e). Bioinks solely composed of microgels have limited stability, making them difficult to use for printing sophisticated structures. To address this problem, another phase of liquid was mixed with microgels to improve printability and morphology fidelity (Fig. 16f) [177]. Furthermore, dynamic covalent bonds could enhance the interaction between microgels while keeping the mechanical moduli of microgels low. Using a microfluidic device, HAMA-phenylboronic acid (PBA) and GelMA were used as the precursor solution to form microgels (Fig. 16g) [194]. Dynamic crosslinker dopamine-modified hyaluronic acid was added to the prepared microgels, where dopamine groups established dynamic covalent bonds with PBA groups located on HAMA to form dynamic cross-linked microgel assembly (DC-MA) bioink. Under the cycle of high and low strain, DC-MA bioink could achieve reversible gel − sol transitions and had excellent self-healing performance. The addition of dynamic crosslinking agents could also increase tissue adhesion, self-healing, and porosity, leading to promoted cell migration and improved wound healing.

Extrusion bioprinting is prevalently used in the preparation of scaffolds. Cao et al. developed a bead-jet printing system that could mass-produce and manipulate microgels loaded with cells via ejecting them at specific locations through nitrogen streams [195]. Their developed technique was conducive to achieving a sparse distribution of microgels with high cell concentration (Fig. 16h). The results showed that compared with the uniform distribution of the same number of cells, regional high cell concentration significantly accelerated the regeneration of skeletal muscle tissue with reduced fibrosis in mice volumetric muscle loss injuries. Recently, an increasing trend in preparing scaffolds by external forces, including laser [196], ultraviolet [197], centrifugation [198], and the formation of scaffolds by interconnecting fixed microgels through the cell growth process, has been reported [199]. A more straightforward approach was recently proposed to 3D print scaffolds with large pores followed by the injection of microgels into the scaffold [200, 201]. In the context of the discussed literature, microgels have exhibited notable efficacy in the encapsulation of cells across a spectrum of applications, underscoring their substantial promise in the realm of cell delivery.

## Future Perspectives and Conclusions

Remarkable progress has been made in all aspects of microgel fabrication and applications to suit the needs of different fields. In particular, the types, materials, devices, and techniques for the fabrication of microgels as well as the loading modes of bioactive substances have recently experienced great improvements. For example, microfluidic techniques allow the highest control over the morphology, complexity, and monodispersity of microgel, especially in solving the limitations of poor monodispersity of emulsification. Based on microfluidic technology, lithography can further increase the capability of the user to create microgels with more complex morphologies and structures. In addition, microfluidic electrospray, centrifugation-based method, and gas-shearing method all avoid using oil and surfactant, greatly improving the bioactivity of cells. As an emerging technology, 3D bioprinting promotes the application of microgels in various fields, such as the development of organoids, providing a new strategy for the development of microgels in the future. In summary, biofabrication techniques with oil-free, surfactant-free, and low mechanical stress along with biomaterials with high cytocompatibility are suitable to generate cell carriers for tissue repair or further build disease models.

The structure and morphology of microgels are very relevant to the types of cells and disease models (Table S1). Many studies have shown that anisotropic structures affect the growth orientation of cells, such as cardiomyocytes, nerve cells, and fibroblasts, that need directional growth in tissues [202–204]. In the tumor disease model, the oriented structure can also guide the migration of cancer cells [202]. In addition, the existence of internal channels in the porous structure makes the exchange of nutrients and metabolic products easier. More importantly, it supports the migration of cells both internally and externally [205–207]. He et al. demonstrated that porous GelMA microspheres can provide a larger space for tumor cell adhesion and growth to simulate the microenvironment of tumor cell growth in vivo [24]. Core–shell microgels and multi-compartment microgels are excellent carriers for the treatment that requires combining two or more kinds of cells. For example, MSCs and epidermal cells were encapsulated in core–shell microgel for hair regeneration [133]. Besides, various natural or modified polymers impart hydrogels’ biochemical and mechanical properties suitable for cell growth [208]. Based on the hydrolytic labile of oxidized SA and the SA stiffness with high molecular weight, microgels could form porous biomaterials inducing new bone growth [209]. Dopamine moieties were grafted into hydrazone-crosslinked hyaluronic acid microgels to increase tissue adhesive properties and demonstrated the proof-of-concept of sutureless implantation in a porcine corneal organ culture model [210]. In summary, microgels of different physicochemical properties can precisely encapsulate cells via various preparation techniques, and simulate the tissue microenvironment. In the future, by adjusting hydrogel materials and the structure of microgels, it is expected to simulate a specific tissue environment, for customizing in vivo models, such as disease models [211].

While some critical issues have been addressed, challenges remain regarding fabrication techniques, hydrogel materials, delivery, and application of microgels. In this section, by providing a roadmap for the future research direction in the field of microgels for biomedical engineering, we highlight several overlooked aspects that need to be more investigated.

**Fig. 17:**
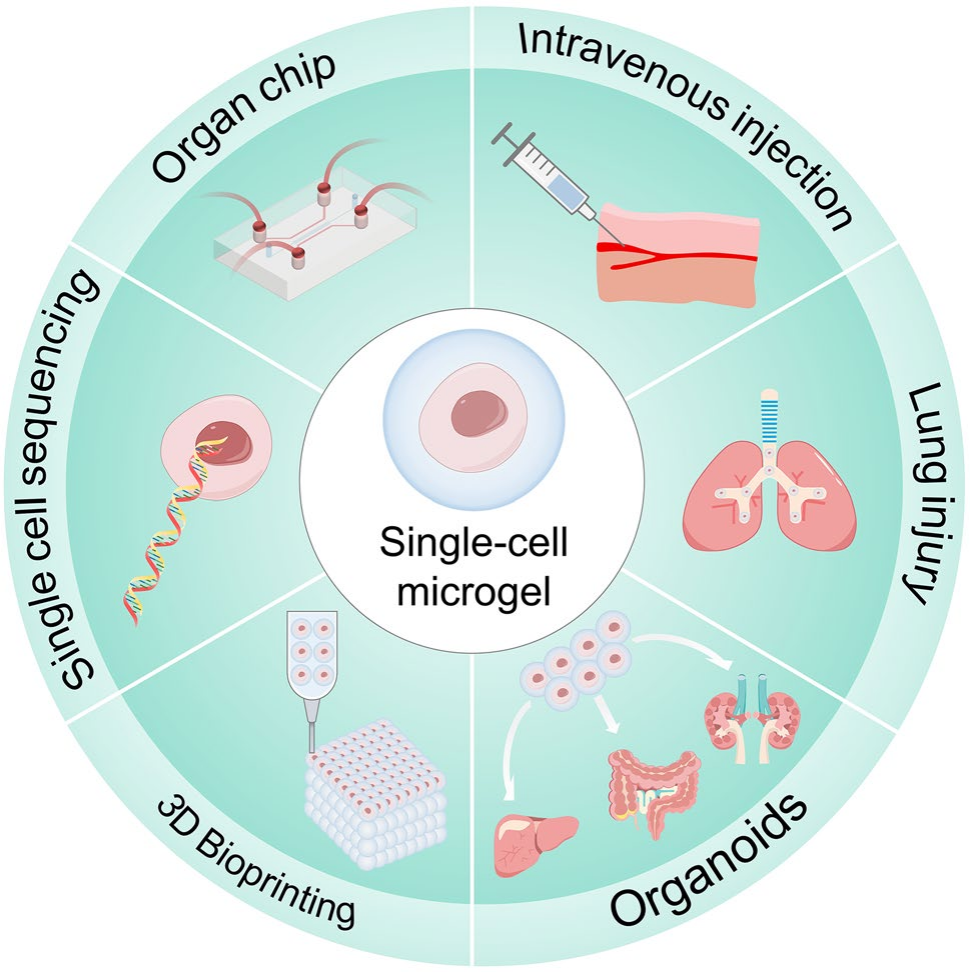
Application of single-cell microgel systems

1. Improvement in fabrication technologies. There is still a substantial need for high-throughput, uniform, and biocompatible strategies for the fabrication of microgels. Cell bioactivity is an important factor in cell therapy. To avoid reduced cell viability during microgel preparation, replacing the oil phase with the incompatible aqueous phase is important. Fabrication of microgels without channel pressure is also key to improving cell viability. Each technology has its own characteristics, and combining different strategies is a potential research direction.
2. Interaction between materials and cell delivery. Biomaterials, such as polysaccharides, proteins, and polymers including natural and synthetic, have been used to develop 3D microcarriers that provide a highly controllable and adaptable platform for cell culture and microtissue formation (Table 2). However, the selection of materials often requires a balance between cell growth adaptability and mechanical properties [28]. For example, SA has excellent mechanical properties, but it does not provide cells with adhesion sites, therefore restricting cell growth. Although cell adhesion can be achieved by grafting the minimal integrin adhesion ligand Arg-Gly-Asp (RGD) on SA, additional chemical reactions are required [23]. On the other hand, GelMA owns RGD and matrix metalloproteinase (MMP) favoring certain cellular activities and tissue restoration. Cells can effectively penetrate GelMA microgels and attach to their network for growth, which may facilitate cell germination but GelMA with low concentrations is hard to self-supporting pattern. Therefore, other materials are generally needed to assist in the generation of GelMA microgels. Recently, many studies have shown that GelMA can be mixed with other materials including biomaterials and nanoparticles [212], to increase its printability and mechanical properties [213]. HAMA was always introduced to GelMA and resulted in enhanced mechanical properties. Besides, hyaluronic acid (HA), a vital element of viscoelastic tissues with high water retention capacity and elasticity, is usually used to deliver MSCs to promote wound healing [214]. In addition to HAMA, bioactive factors such as DNA, micro-ribonucleic acid (miRNA), cytokines, and exosomes can be also mixed with materials to enhance the mechanical and therapeutic properties of the materials [215, 216]. In addition, the targeting ability of material facilitates the accumulation of cells at the defect site and enhances the therapeutic effect. Based on the colon-targeted property of SA and chitosan, the microgels can accumulate well in the colon [89, 217]. To further meet the requirements of tissue repair and regenerative medicine applications, more biomaterials with excellent biocompatibility and mechanical properties need to be developed in the future.
3. Clinical transformation. Academic research needs to be fully transformed into practical and clinical applications. With the current preparation technology, although functional microgels with complex structures can be readily prepared and have excellent therapeutic effects in animal experiments, most of them have not been further studied in clinical trials.
4. Diversity of delivery. Although microgels have successfully hosted cells, cellular secretions, and drugs, there is rarely research on encapsulating microorganisms. Therefore, the encapsulation of microorganisms and other bioactive substances and investigating their applications is another research direction in the field of microgels.
5. Microgels for organoids. Human organs are jointly maintained and grown via a variety of cells. Up to now, Matrigel has become an important carrier in organoid culture, but it also faces many limitations such as prolonged culturing time, high economic cost, poor homogeneity, and large volume of organoids which easily leads to central necrosis. To overcome these problems, microgels-organoids are proposed [218, 219]. Owing to their multi-compartment porous structures and high biocompatibility, microgels are excellent carriers for the co-culture of a variety of cells. They have received extensive attention in the field of organoids and microtissue regeneration, but still, many problems need to be addressed. Although large-scale microgels can be prepared to grow larger organoids, the limitations in nutrients and metabolite transport in the central area remain. Therefore, the formation of 3D tissue spheroids using multiple small-scale microgels as micro-units to mimic native tissue microenvironments can be a potential subject of research. 3D cell aggregates formed by microgels are more conducive to the cultivation of organoids with complex functions and larger volumes. One important advantage of microgel-based models is the cellular capability to secrete ECM components with effective communication between cells in a mimetic microenvironment.
6. Single cell in microgels. Microgels with small sizes encapsulating single-cell have the feasibility of direct intravenous delivery and are easier to reach small functional units such as alveoli. In addition, the increase of specific surface area enhances the rapid diffusion of oxygen, nutrients, and cellular waste, avoiding unwanted outcomes such as cell hypoxia and infarction after transplantation. However, preparation of microgels and cell encapsulation on such a small scale pose great challenges. Cell encapsulation and the study of single cells are an important development direction in the future. The application areas of single-cell microspheres, including organoids, lung injury, 3D bioprinting, etc. are shown in Fig. 17.

**Table 2:** Hydrogel materials for fabricating microgels

	Materials	Crosslinking method	References
Natural	Alginate	Cross-linked with calcium ions	[14, 15, 65]
	CMC	Cross-linked with cupric/iron ions	[220–222]
	Gelatin	Cross-linked by microbial transglutaminase/Genipin	[152, 174, 223]
	CS	Cross-linked by electrostatic interactions/Genipin	[171, 224, 225]
	Silk fibroin	UV crosslinking	[106, 226, 227]
	Agarose	Cross-linked by crosslinker	[228–230]
	Matrigel	Cross-linked by temperature	[195, 231, 232]
Synthetic	GelMA	UV crosslinking	[74, 133, 233]
	HAMA	UV crosslinking	[176, 195, 234]
	PEG	UV crosslinking	[63, 190, 235]
	PEO	Cross-linked by temperature or UV	[236–238]
	PVA	Cross-linked by crosslinker or UV	[26, 239, 240]
	PLGA	Solvent evaporation	[141, 241, 242]

*CMC* carboxymethyl cellulose, *CS* chitosan, *GelMA* gelatin methacryloyl, *HAMA* hyaluronic acid methacryloyl, *PEG* poly (ethylene glycol), *PEO* poly (ethylene oxide), *PVA* poly (vinyl alcohol), *PLGA* poly (lactic-co-glycolic acid), *UV* ultraviolet

In conclusion, the microgel fabrication techniques were thoroughly discussed. Various strategies for preparing complex microgels, such as Junus, core–shell, multi-compartment, and porous, were introduced and elaborated in detail. We demonstrated how microgels are excellent carriers for different types of cells such as stem cells, islet cells, cardiac cells, and Schwann cells. They can not only improve the retention and viability of cells but also induce differentiation and co-delivery with other bioactive substances, such as growth factors and exosomes. As one of the components of 3D printing bioinks, microgels are also used in the fabrication of scaffolds. With the help of microgels, it is expected to improve the printability of various bioinks and achieve higher printing resolution. Overall, there are important developments ahead in the field of microgels to pave the way for their clinical applications in tissue engineering and regenerative medicine.

## Supplementary Information

Below is the link to the electronic supplementary material.

Supplementary file1 (DOCX 96 kb)
