# Supplementary material for: Microgels for Cell Delivery in Tissue Engineering and Regenerative Medicine
Source: Nanomicro Lett. 2024 Jun 17;16:218. doi: 10.1007/s40820-024-01421-5 (PMC11183039; doi:10.1007/s40820-024-01421-5)
Supplement: Supplementary file 1 — Supplementary file1 (DOCX 96 kb) [file 40820_2024_1421_MOESM1_ESM.docx]

Supporting Information for

**Microgels for Cell Delivery in Tissue Engineering and Regenerative Medicine**

Leyan Xuan^1,#^, Yingying Hou^1,#^, Lu Liang^1,#^, Jialin Wu^1^, Kai Fan^2^, Liming Lian^4^, Jianhua Qiu^1^, Yingling Miao^1^, Hossein Ravanbakhsh^3,^*, Mingen Xu^2,^*, Guosheng Tang^1,^*

^1^ Guangzhou Municipal and Guangdong Provincial Key Laboratory of Molecular Target & Clinical Pharmacology, the NMPA and State Key Laboratory of Respiratory Disease, School of Pharmaceutical Sciences and the Fifth Affiliated Hospital, Guangzhou Medical University, Guangzhou 511436, P. R. China

^2^ School of Automation, Hangzhou Dianzi University, Hangzhou 310018, P. R. China

^3^ Department of Biomedical Engineering, The University of Akron, Akron, OH 44325, USA

^4^ Wallace H. Coulter Department of Biomedical Engineering, Georgia Institute of Technology, Atlanta, Georgia 30332, USA

^#^ Leyan Xuan, Yingying Hou, and Lu Liang contributed equally to this work.

*Corresponding authors. E-mail: [guoshengtang@gzhmu.edu.cn](mailto:guoshengtang@gzhmu.edu.cn) (Guosheng Tang), xumingen@hdu.edu.cn (Mingen Xu), or hravanbakhsh@uakron.edu (Hossein Ravanbakhsh)

**Supplementary Figures and Tables**


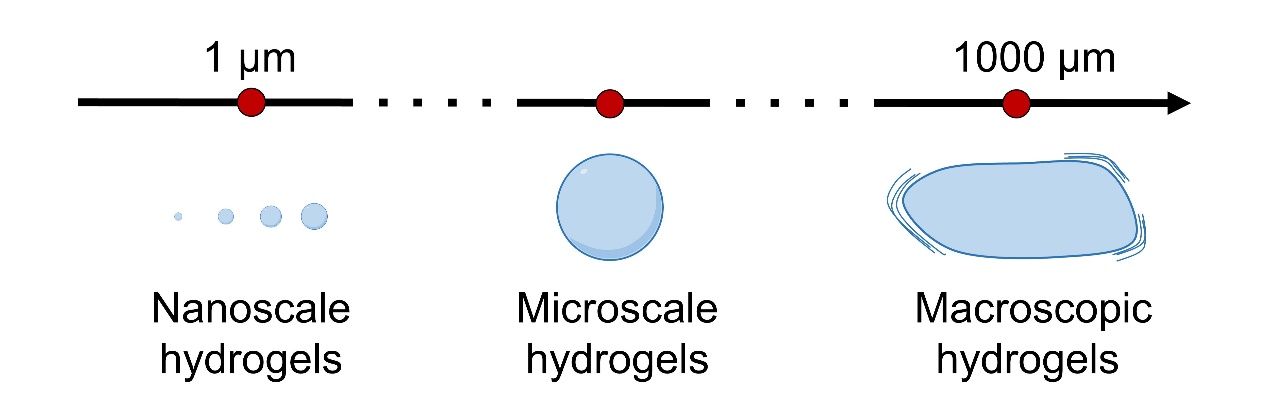


**Fig. S1** Schematic of nanoscale hydrogels, microscale hydrogels and macroscopic hydrogels

**Table S1** Different types of microgels for cells delivery and their applications

| **Types of microgels** | **cell delivery types** | **applications** |
| --- | --- | --- |
| Homogenous | eUCB-MSCs [S1], Islet cell [S2], HEK293T cell [S3], hESCs and HUVECs [S4], hiPSCs [S5, S6], MSCs [S7], hDPSCs [S8] | Osteochondral structures biofabrication [S1]; immune protection [S2]; vascular regeneration [S3]; Efficient hepatic differentiation [S4]; Engineered cardiac tissue production [S5]; Bone Repair [S7]; endodontic regeneration [S8]; |
| Core-shell | BMSCs [S9], MSCs and EPCs [S10], hiPSCs [S11] | Bone Repair [S9]; hair regeneration [S10]; islet organoids formation [S11]; |
| Janus | MDA-MB-231 [S12], MSCs [S13], fibroblasts, ECs [S14], | Microtissue production [S12]; cartilage repair [S13]; heterogeneous tissues simulation [S14]; |
| Multi-compartment | HepG_2_, Hela [S15], HepG_2_, HUVECs [S16], EA.hy926, HepG_2_/C3A [S17] | Tissue models construction [S15, S16]; hepatic-lobule-like structure fabrication [S17]; |
| Porous | ADSCs [18, 19], MSCs [S20–S22], C2C12 [S23], | Ischemic limb regeneration [S18]; disc degeneration repair [S19]; acute liver failure recovery [S20]; cartilage regeneration [S21]; systemic lupus erythematosus treatment [S22]; vascularization [S23] |

eUCB-MSCs, equine umbilical cord blood-derived MSCs; hESCs, human embryonic stem cells; HUVECs, human umbilical vein endothelial cells; hiPSCs, human induced pluripotent stem cells; MSCs, mesenchymal stem cells; hDPSCs, EPCs, human dental pulp stem cells, epidermal cells; C2C12, mouse skeletal myoblasts cells; ECs, endothelial cells; HepG_2_, human hepatocarcinoma; ADSCs, adipose-derived stem cells; C2C12, mouse skeletal myoblasts.

**Supplementary References**

1. X. Cui, C. R. Alcala-Orozco, K. Baer, J. Li, C. A. Murphy et al., 3d bioassembly of cell-instructive chondrogenic and osteogenic hydrogel microspheres containing allogeneic stem cells for hybrid biofabrication of osteochondral constructs. Biofabrication **14**, 034101 (2022). <https://doi.org/10.1088/1758-5090/ac61a3>
2. M. A. Bochenek, O. Veiseh, A. J. Vegas, J. J. McGarrigle, M. Qi et al., Alginate encapsulation as long-term immune protection of allogeneic pancreatic islet cells transplanted into the omental bursa of macaques. Nat. Biomed. Eng. **2**, 810-821 (2018). <https://doi.org/10.1038/s41551-018-0275-1>
3. J. Shen, Y. Ji, M. Xie, H. Zhao, W. Xuan et al.,Cell-modified bioprinted microspheres for vascular regeneration. Mater. Sci. Eng. C. Mater. Biol. Appl. **112**, 110896 (2020). <https://doi.org/10.1016/j.msec.2020.110896>
4. S. Deng, X. Zhao, Y. Zhu, N. Tang, R. Wang et al., Efficient hepatic differentiation of hydrogel microsphere-encapsulated human pluripotent stem cells for engineering prevascularized liver tissue. Biofabrication **15**, 015016 (2022). <https://doi.org/10.1088/1758-5090/aca79b>
5. F. B. Finklea, Y. Tian, P. Kerscher, W. J. Seeto, M. E. Ellis et al., Engineered cardiac tissue microsphere production through direct differentiation of hydrogel-encapsulated human pluripotent stem cells. Biomaterials **274**, 120818 (2021). <https://doi.org/10.1016/j.biomaterials.2021.120818>
6. I. Gal, R. Edri, N. Noor, M. Rotenberg, M. Namestnikov et al., Injectable cardiac cell microdroplets for tissue regeneration. Small **16**, e1904806 (2020). <https://doi.org/10.1002/smll.201904806>
7. X. Zhao, S. Liu, L. Yildirimer, H. Zhao, R. Ding et al., Injectable stem cell-laden photocrosslinkable microspheres fabricated using microfluidics for rapid generation of osteogenic tissue constructs. Adv. Funct. Mater. **26**, 2809-2819 (2016). https://doi.org/10.1002/adfm.201504943
8. Q. Zhang, T. Yang, R. Zhang, X. Liang, G. Wang et al., Platelet lysate functionalized gelatin methacrylate microspheres for improving angiogenesis in endodontic regeneration. Acta Biomater. **136**, 441-455 (2021). <https://doi.org/10.1016/j.actbio.2021.09.024>
9. L. Yang, Y. Liu, L. Sun, C. Zhao, G. Chen et al., Biomass microcapsules with stem cell encapsulation for bone repair. Nano-Micro Lett. **14**, 4 (2021). <https://doi.org/10.1007/s40820-021-00747-8>
10. J. Huang, D. Fu, X. Wu, Y. Li, B. Zheng et al., One-step generation of core-shell biomimetic microspheres encapsulating double-layer cells using microfluidics for hair regeneration. Biofabrication **15**, 025007 (2023). <https://doi.org/10.1088/1758-5090/acb107>
11. H. Liu, Y. Wang, H. Wang, M. Zhao, T. Tao et al., A droplet microfluidic system to fabricate hybrid capsules enabling stem cell organoid engineering. Adv. Sci. **7**, 1903739 (2020). <https://doi.org/10.1002/advs.201903739>
12. Y.C. Lu, W. Song, D. An, B.J. Kim, R. Schwartz et al., Designing compartmentalized hydrogel microparticles for cell encapsulation and scalable 3d cell culture. J. Mater. Chem B **3**, 353-360 (2015). <https://doi.org/10.1039/c4tb01735h>
13. R. G. Thomas, A. R. Unnithan, M. J. Moon, S. P. Surendran, T. Batgerel et al., Electromagnetic manipulation enabled calcium alginate janus microsphere for targeted delivery of mesenchymal stem cells. Int. J. Biol. Macromol. **110**, 465-471 (2018). <https://doi.org/10.1016/j.ijbiomac.2018.01.003>
14. H. Zheng, W. Du, Y. Duan, K. Geng, J. Deng, C. Gao, Biodegradable anisotropic microparticles for stepwise cell adhesion and preparation of janus cell microparticles. ACS. Appl. Mater. Interfaces **10**, 36776-36785 (2018). <https://doi.org/10.1021/acsami.8b14884>
15. G. Tang, R. Xiong, D. Lv, R. X. Xu, K. Braeckmans et al., Gas-shearing fabrication of multicompartmental microspheres: A one-step and oil-free approach. Adv. Sci. **6** , 1802342 (2019). <https://doi.org/10.1002/advs.201802342>
16. H. Wang, H. Liu, F. He, W. Chen, X. Zhang et al., Flexible generation of multi‐aqueous core hydrogel capsules using microfluidic aqueous two‐phase system. Adv. Mater. Technol. **5**, 2000045 (2020). <https://doi.org/10.1002/admt.202000045>
17. G. Hong, J. Kim, H. Oh, S. Yun, C. M. Kim et al., Production of multiple cell-laden microtissue spheroids with a biomimetic hepatic-lobule-like structure. Adv. Mater. **33**, e2102624 (2021). <https://doi.org/10.1002/adma.202102624>
18. R. Wang, F. Wang, S. Lu, B. Gao, Y. Kan et al., Adipose-derived stem cell/fgf19-loaded microfluidic hydrogel microspheres for synergistic restoration of critical ischemic limb. Bioact. Mater. **27**, 394-408 (2023). <https://doi.org/10.1016/j.bioactmat.2023.04.006>
19. H. Xu, M. Sun, C. Wang, K. Xia, S. Xiao et al., Growth differentiation factor-5-gelatin methacryloyl injectable microspheres laden with adipose-derived stem cells for repair of disc degeneration. Biofabrication **13**, 015010 (2020). <https://doi.org/10.1088/1758-5090/abc4d3>
20. J. Wang, D. Huang, H. Yu, H. Ren, L. Shang, Biohybrid response microparticles decorated with trained-mscs for acute liver failure recovery. Adv. Healthc. Mater. **11**, e2201085 (2022). <https://doi.org/10.1002/adhm.202201085>
21. X. Li, X. Li, J. Yang, J. Lin, Y. Zhu et al., Living and injectable porous hydrogel microsphere with paracrine activity for cartilage regeneration. Small **19**, e2207211 (2023). <https://doi.org/10.1002/smll.202207211>
22. G. C. Min Nie, Cheng Zhao, Jingjing Gan, Mihribangvl Alip, Yuanjin Zhao et al., Bio-inspired adhesive porous particles with human mscs encapsulation for systemic lupus erythematosus treatment. Bioact. Mater. **10**, 84-90 (2021). <https://doi.org/10.1016/j.bioactmat.2020.07.018>
23. R. K. Kankala, J. Zhao, C. G. Liu, X. J. Song, D. Y. Yang et al., Highly porous microcarriers for minimally invasive in situ skeletal muscle cell delivery. Small **15**, e1901397 (2019). <https://doi.org/10.1002/smll.201901397>
